# Heterogeneity of tyrosine-based melanin anabolism regulates pulmonary and cerebral organotropic colonization microenvironment of melanoma cells

**DOI:** 10.7150/thno.69198

**Published:** 2022-01-31

**Authors:** Xuefeng Wang, Yu Chen, Bin Lan, Yu Wang, Wansong Lin, Xu Jiang, Jiayin Ye, Bingxue Shang, Chao Feng, Jun Liu, Jingjie Zhai, Muhan Xu, Qing Li, Liangyu Lin, Mingyuan Hu, Fanjun Zheng, Ling Chen, Changshun Shao, Ying Wang, Yufang Shi

**Affiliations:** 1 The Third Affiliated Hospital of Soochow University and State Key Laboratory of Radiation Medicine and Protection, Institutes for Translational Medicine, Soochow University, 199 Renai Road, Suzhou, Jiangsu 215123, China.; 2 CAS Key Laboratory of Tissue Microenvironment and Tumor, Shanghai Institute of Nutrition and Health, University of Chinese Academy of Sciences, Chinese Academy of Sciences, 320 Yueyang Road, Shanghai 200031, China.; 3 Fujian Provincial Key Laboratory of Translational Cancer Medicine, Cancer Bio-immunotherapy Center, Fujian Medical University Cancer Hospital, 420 Fuma Road, Fuzhou 350014, China.; 4 Shanghai Jiao Tong University School of Medicine, Shanghai Center for Systems Biomedicine Research, Shanghai Jiao Tong University, 800 Dongchuan Road, Shanghai 200240, China.; 5 Department of Experimental Medicine, TOR, University of Rome Tor Vergata, Rome 00133, Italy.

**Keywords:** Melanoma, organotropic colonization, tumor heterogeneity, melanin anabolism, immune checkpoint

## Abstract

**Background:** Dietary tyrosine regulating melanoma progression has been well-recognized. However, whether tyrosine-based melanin anabolism contributes to pulmonary and cerebral organotropic colonization of melanoma remains elusive. Furthermore, approaches based on targeting tyrosinase activity to inhibiting multi-organ metastasis of melanoma cells need to be designed and validated.

**Methods:** Patients derived melanoma cells and mouse B16 melanoma cells with different pigmentation were employed in this investigation. Tyrosine content dynamics in tumors and multiple organs during the melanoma progression was monitored, and tyrosine-based melanin synthesis of melanoma cells derived from multi-organ was determined. Additionally, we also adopted RNA-seq, flow cytometry, real-time PCR and composite metastasis mouse model to analyze organotropic colonization and to validate designed therapeutic strategies.

**Results:** B16 melanoma cells with high activity of tyrosinase and sensitivity of tyrosine utilization for melanin synthesis (Tyr-H cells) easily colonized in the lung, while B16 melanoma cells lacking above characteristics (Tyr-L cells) exhibited potent proliferation in the brain. Mechanistically, Tyr-H cells recruited and trained neutrophils and macrophages to establish pulmonary metastatic niche dependent on highly secreted CXCL1 and CXCL2 and an excessive melanosome accumulation-induced cell death. Tyr-L cells enhanced PD-L1 expression in tumor-infiltrated macrophages when they are progressing in the brain. Accordingly, intervention of tyrosinase activity (2-Ethoxybenzamide or hydroquinone) in combination with inhibitors of phagocytosis (GSK343) or chemotaxis (SB225002) suppressed organotropic colonization and significantly improved the survival of melanoma- bearing mice treated with immune checkpoint blockade (PD1 antibody).

**Conclusions:** The heterogeneity of melanoma cells in utilization of tyrosine is associated with organotropic colonization, providing the basis for developing new strategies to combat melanoma.

## Introduction

Malignant melanoma is the most lethal skin cancer with constantly increasing incidence worldwide. Diverse mutations contribute to intratumor heterogeneity and are associated with varying metastatic potential to different organs 1. Clinically evident metastasis is an ominous sign in melanoma patients, and about 95% of patients with three or more distant metastatic sites die within one year 2. Metastatic melanoma can be found in multiple organs, mainly in the lung and brain, followed by liver and bone 3-5. However, the factors determining organotropic metastasis of melanoma remain elusive.

Melanoma is the most common in light-skinned people, but it can also afflict those with darker pigment 6. During the progression of melanomas, loss of pigment often occurs, and the coloration of melanoma cells is associated with the prognosis of melanoma. In some reports, amelanotic melanoma exhibited more aggressive pathologic features and poorer melanoma-specific survival than pigmented melanoma 7-9. In another report, melanomas produced melanin to protect themselves from radiotherapy 10. Using melanotic MNT-1 cells enriched with mature stage III and IV melanosomes and SK-MEL-28 cells with immature stage I and II melanosomes, studies have demonstrated that MNT-1 cells proliferate faster and are more sensitive to cisplatin than SK-MEL-28 cells 11. Therefore, pigment may regulate the metastasis behavior and response to therapies in melanoma cells.

Melanin is synthesized enzymatically from L-tyrosine (hereinafter referred to as tyrosine) and L-dihydroxyphenylalanine (L-DOPA), or non-enzymatically from dopamine 12-15. Among them, tyrosine not only serves as the substrate but also as a regulator of melanogenesis and cellular functions 12, 14, even in Bomirski Ab amelanotic melanoma cells 13. Of note, dopamine is not a precursor to cutaneous melanin, it is a neurotransmitter that can autoxidize to neuromelanin 15. Importantly, the neuroendocrine activity of melanocytes has been proposed by Dr. Andrzej Slominski and his colleagues, the metabolism of melanocytes does not only coordinate cutaneous but can also affect whole-body homeostasis 16. Enhanced tyrosine consumption can be observed in melanoma patients, when melanoma developed to the stage II ~ stage III, L-DOPA, one metabolite of tyrosine, was significantly upregulated 17, and when the disease developed to the stages III ~ IV, strong melanogenesis significantly shorten the overall and disease-free survival time 18. Restriction on the utilization of tyrosine/phenylalanine, methionine or glutamine in human derived A375 and MeWo melanoma cells can impair the invasive ability of these melanoma cells through disturbing the interaction of GTPase and Ras/Ros 19. In addition, restriction of dietary tyrosine and phenylalanine improved the chemotherapy efficacy of melanoma 20, 21. Therefore, detailed investigations of the properties of melanoma cells in utilizing tyrosine and other metabolites will help to decipher the cellular behavior of melanoma.

We found that murine B16 melanoma exhibited heterogeneity in tyrosine utilization. Cells with higher *Tyr* expression (Tyr-H cells) were apt to use tyrosine to synthesize melanin and preferred to localize and grow in the lung, while cells showing lower *Tyr* expression (Tyr-L cells) were insensitive to tyrosine and more aggressive in the brain. These results suggest that the utilization of tyrosine by melanoma cells is related to their organotropic colonization. Such difference was related to the distinct abilities of Tyr-H and Tyr-L melanoma cells in shaping tumor immune microenvironment. Targeting tyrosine utilization, together with inhibition of phagocytosis or chemotaxis process, can suppress organotropic colonization of melanoma cells and significantly improve the survival of tumor bearing mice.

## Results

### Tyrosine-based melanin synthesis affects organotropic colonization of melanoma cells

**Figure 1:**
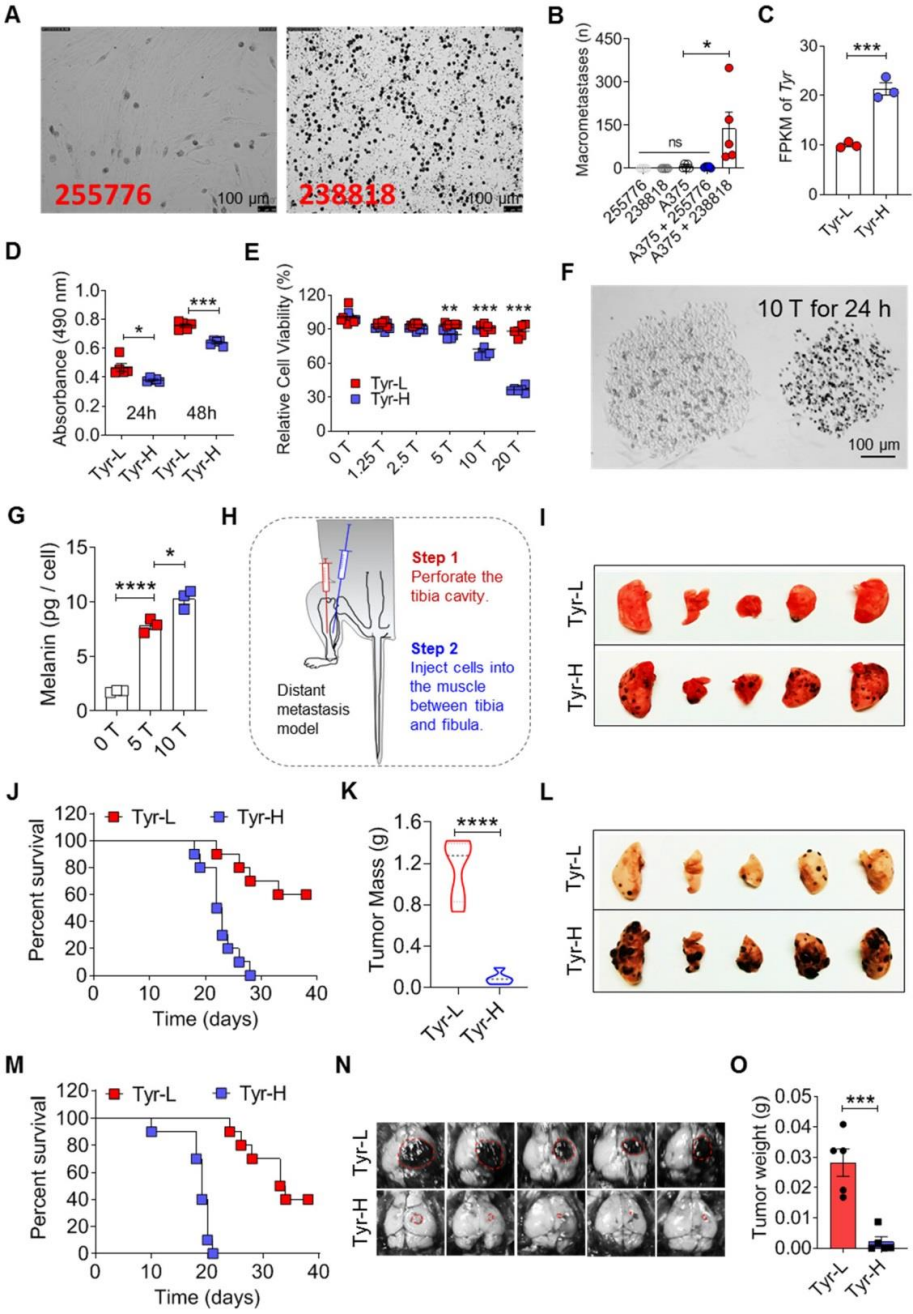
** The heterogeneity of tyrosine-based melanin synthesis affects organotropic colonization of melanoma cells. A.** Microscope photos of melanoma cells derived from patients. Left photo (255776) showed amelanotic melanoma cells and right photo (238818) showed pigment melanoma cells. Scale bar is 100 μm. **B.** Human melanoma cell line A375 cells were mixed with 255776 cells or 238818 cells and co-transplanted into NOD-SCID mice by *i.v.* injection. The pulmonary macrometastases were counted on day 30. Injection of 255776 cells, 238818 cells or A375 alone as the control. **C.** *Tyr* gene expression level measured by RNA sequencing technology. **D-E.** Relative cell viability was evaluated by MTS assay. Tyr-H cells and Tyr-L cells were respectively cultured in the standard medium for 24 and 48 h (D), or in the conditional medium with different concentration of tyrosine (0, 1.25, 2.5, 5, 10 and 20 times higher than the standard concentration, 0 T, 1.25 T, 2.5 T, 10 T, 20 T) for 24 h (E). **F.** Representative microscope photos of Tyr-L cell clone (left) and Tyr-H cell clone (right) under the 10 T tyrosine treatment for 24 h. **G.** Absolute intracellular accumulation of melanin in the Tyr-H cells. Tyr-H cells were treated with 5 T and 10 T tyrosine for 30 h. **H-K.** Schematic showing a distant metastasis model of melanoma (H). Representative results of the lung with metastasis lesions (I), percent survival (J), and mass of tumor in muscle (K). **L-M.** Tyr-L cells and Tyr-H cells were injected intravenously. Representative photos of the lung with metastasis lesions (L), and percent survival (M). **N-O**. Photos and mass of the melanoma focus localized in brain from experimental cerebral metastasis model. All data given as mean ± SEM., n ≥ 3. *P < 0.05, **P < 0.01, ***P < 0.001, ****P < 0.0001, ns, not significant. All results shown are representative of three independent experiments.

During the growth of B16 melanoma, tyrosine content ratios were significantly decreased in the tumor, lung, and brain, but increased in the liver and pancreas (Figure S1A-C). Of note, the tyrosine concentrations (~1,000 ng/g) in the lung and brain were comparable to those of tumor in small size (less than 1,000 mm^3^, figure S1A). In a model of systemic metastatic melanoma constructed by cardiac injection, we observed that B16 cells were engrafted and grew in the gonadal fat, lung, liver, muscle and other organs (Figure S1D). In comparison to B16 cells isolated from these tissues and cultured *in vitro*, B16 cells derived from lung were the most apt to utilize tyrosine for melanin synthesis (Figure S1E). These results indicate that the efficiency of tyrosine utilization by B16 cells and the abundance of tyrosine in the tissues may affect the colonization of B16 cells. Indeed, not all patient-derived primary tumor cells and human tumor cell lines have strong tumor-forming ability in immunodeficient mice. When A375 cells, a human melanoma cell line, were co-injected with patient-derived pigmented melanoma cells (238818), tumor was observed in the lung. However, A375 cells alone or co-injected with patient-derived amelanotic melanoma cells (255776) failed to form tumor in the lung (Figure 1A-B). *Tyr* gene encoding tyrosinase controls tyrosine-based melanin anabolism. We determined the expressions of MITF, Tyrosinase, TYRP1 and TYRP2, which are involved in tyrosine-based melanogenesis 22-25, measured tyrosinase activity and utilization of tyrosine for melanin synthesis in B16 cell subclones, and divided them into high (Tyr-H) and low (Tyr-L) utilization of tyrosine, respectively (Figure 1C, and figure S2). Tyr-H cells showed stronger tyrosinase activity and enhanced Tyrosinase, TYRP1 and TYRP2 expression without difference in MITF expression (Figure S2A-D and S2I-J). Additionally, both tyrosinase agonist 2-ETZ and tyrosinase substrate L-tyrosine significantly increased MITF, Tyrosinase, TYRP1 and TYRP2 expression and tyrosinase activity in parental B16 melanoma cells (Figure S2E-H and S2K-L).

Tyr-L cells proliferated faster than Tyr-H cells with or without tyrosine addition (Figure 1D-E and figure S3A-E). Upon exposure to tyrosine, melanin production was increased in Tyr-H cells (Figure 1F-G). Because the therapeutic efficacy on distant metastatic tumor may depend on the presence of *in situ* tumor, a composite model with *in situ* tumor and distant metastasis is needed to study tumor progression and anti-tumor mechanism 26. We designed and established a composite melanoma model with* in situ* tumor in the muscle and the tumor that spontaneous metastasis to the lung (Figure 1H). In this model, Tyr-H cells, but not Tyr-L cells, dominantly metastasized to the lung (Figure 1I). Although mice bearing Tyr-H melanoma exhibited less tumor burden in the muscle, their life span was greatly shortened (Figure 1J-K). The experimental lung metastasis model further confirmed that Tyr-H cells could more easily colonize in the lung than Tyr-L cells (Figure 1L-M). We wondered if the tyrosine-rich pulmonary microenvironment contributes to the chemotaxis of Tyr-H cells to the lung. A transwell experiment, however, did not reveal an increased migration of Tyr-H cells than Tyr-L cells upon tyrosine treatment (Figure S3F-J), indicating that other mechanisms could contribute to the preferential colonization of Tyr-H cells in the lung.

Brain is another organ that melanoma can metastasize easily 27. We found that the tyrosine concentration in the brain is close to that of small *in situ* melanoma tumors (Figure S1A). Interestingly, when we used stereotactic intracranial injection to construct a brain graft model of melanoma, we found that Tyr-L cells formed much larger tumors (Figure 1N-O and figure S3K). Like Tyr-L cells, B78H1, an amelanotic clone derived from the murine melanoma B16 in the laboratory of Selma Silagi 28, lacked the ability to utilize tyrosine for melanin synthesis and to colonize the lung (Figure S3L). However, these B78H1 cells formed light-colored tumor in the brain (Figure S3M). Based on the color of the melanoma colonized in the brain (Figure S3M), we speculated that Tyr-L cells can use dopamine to synthesize melanin, but the dopamine-based melanin synthesis did not affect their brain colonization. These data demonstrate that the heterogeneity in tyrosine utilization in melanoma affects their organotropic colonization.

### Properties of tyrosine resistant B16 cells *in vitro* and* in vivo*

**Figure 2:**
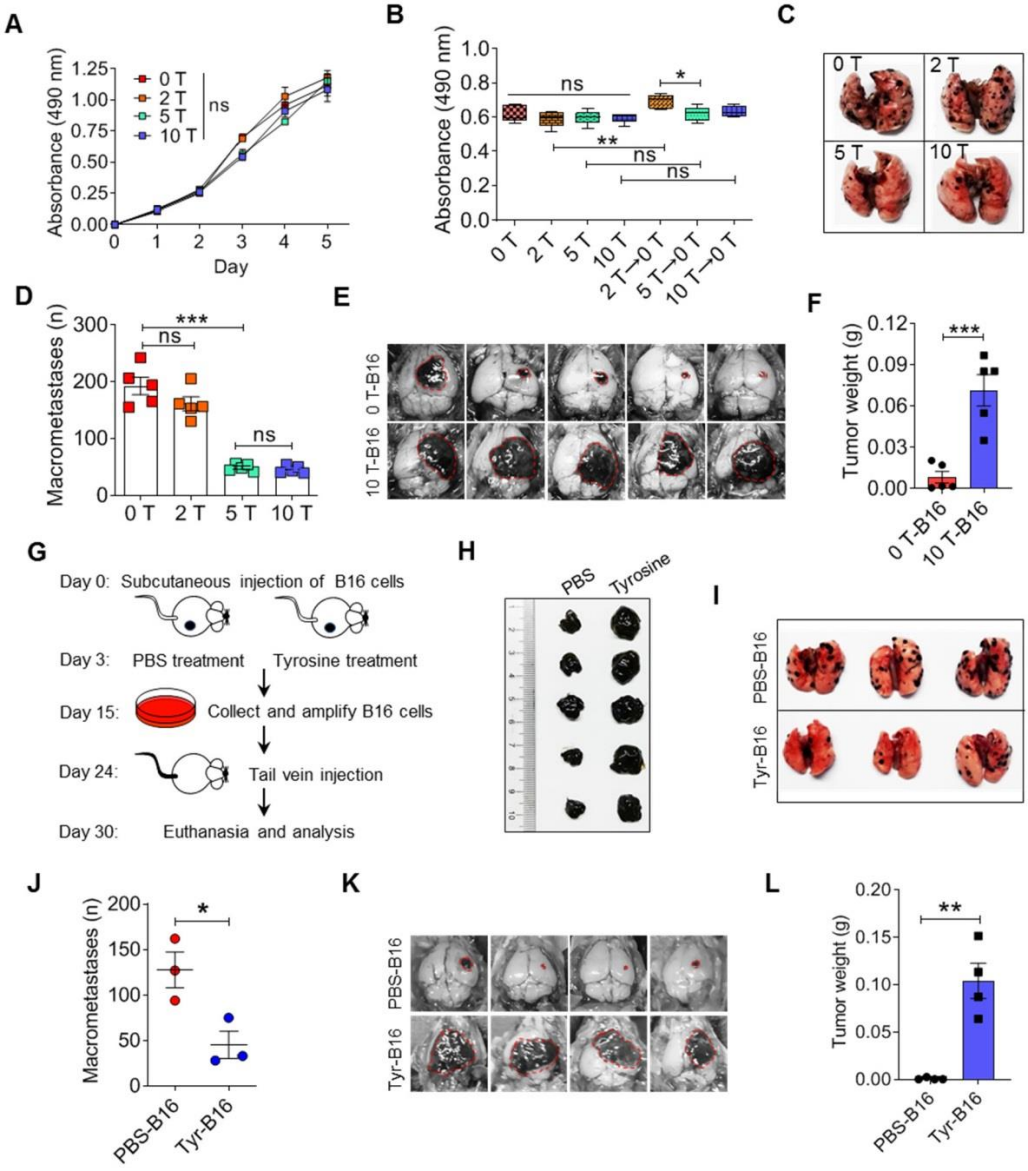
** Malignant behaviors of tyrosine-resistant melanoma cells *in vitro* and *in vivo*. A-B.** Relative cell viability was measured by MTS assay. The same number of tyrosine trained cells were cultured in the fine-tuned medium with different tyrosine addition for 48 h (A), or in the standard medium for 6 days (B). **C-D.** Representative photos and counts of the lung with metastasis lesions from experimental pulmonary metastasis model on day 15. Normal (0 T-), 2 T- , 5 T- and 10 T- B16 cells were injected intravenously. **E-F.** Photos and mass of the melanoma focus in brain from experimental cerebral metastasis model. **G-H.** Schematic showing Tyr-L cells enrichment procedure *in vivo* (G). Photos of B16 subcutaneous tumor. Tumor bearing mice were treated with intratumor injection of tyrosine (H). **I-J.** Photos and counts of the lung with metastasis lesions from experimental pulmonary metastasis model. Normal B16 cells (PBS-B16) and tyrosine-enriched B16 (Tyr-B16) cells were injected intravenously. **K-L.** Photos and mass of the brain with metastasis lesions from experimental cerebral metastasis model. All data given as mean ± SEM., n ≥ 3. *P < 0.05, **P < 0.01, ***P < 0.001, ns, not significant. All results shown are representative of three independent experiments.

The mutagen ethylmethanesulfonate can induce amelanotic variants that can be isolated from wild-type melanotic melanoma cells, and these amelanotic variants lost tyrosinase activity 29. We established a new method for selecting amelanotic B16 cells. When high doses of tyrosine were added into the culture medium, partially sensitive B16 cells would be killed (these cells had similar characteristics to the Tyr-H cells established above), leading to survival and enrichment of insensitive cells (these cells have similar characteristics to the Tyr-L cells established above). After 20 passages, 2-fold, 5-fold and even 10-fold higher than standard concentration of tyrosine (abbreviated as 2 T, 5 T and 10 T) in the medium did not reduce the cell viability and proliferation of the remaining B16 cells with low tyrosine utilization (Figure 2A-B and figure S4A-C). Removal of tyrosine in culture medium promoted the cell viability in the 2 T tyrosine-resistant group (Figure 2B). Interestingly, the pulmonary colonization of 5 T- and 10 T- tyrosine resistant B16 cells was significantly decreased (Figure 2C-D). However, they grew in brain easily (Figure 2E-F). We next injected tyrosine into subcutaneous B16 tumors to select tyrosine-resistant cells *in vivo* (Figure 2G-H). Similarly, tyrosine-selected B16 cells showed increased growth in the brain, but had impaired colonization in the lung (Figure 2I-L). Therefore, exposure to excess tyrosine facilitates the selection of Tyr-L cells that are more apt to grow in the brain.

### Tyr-H cells recruit neutrophils and macrophages to construct pulmonary metastatic niche

**Figure 3:**
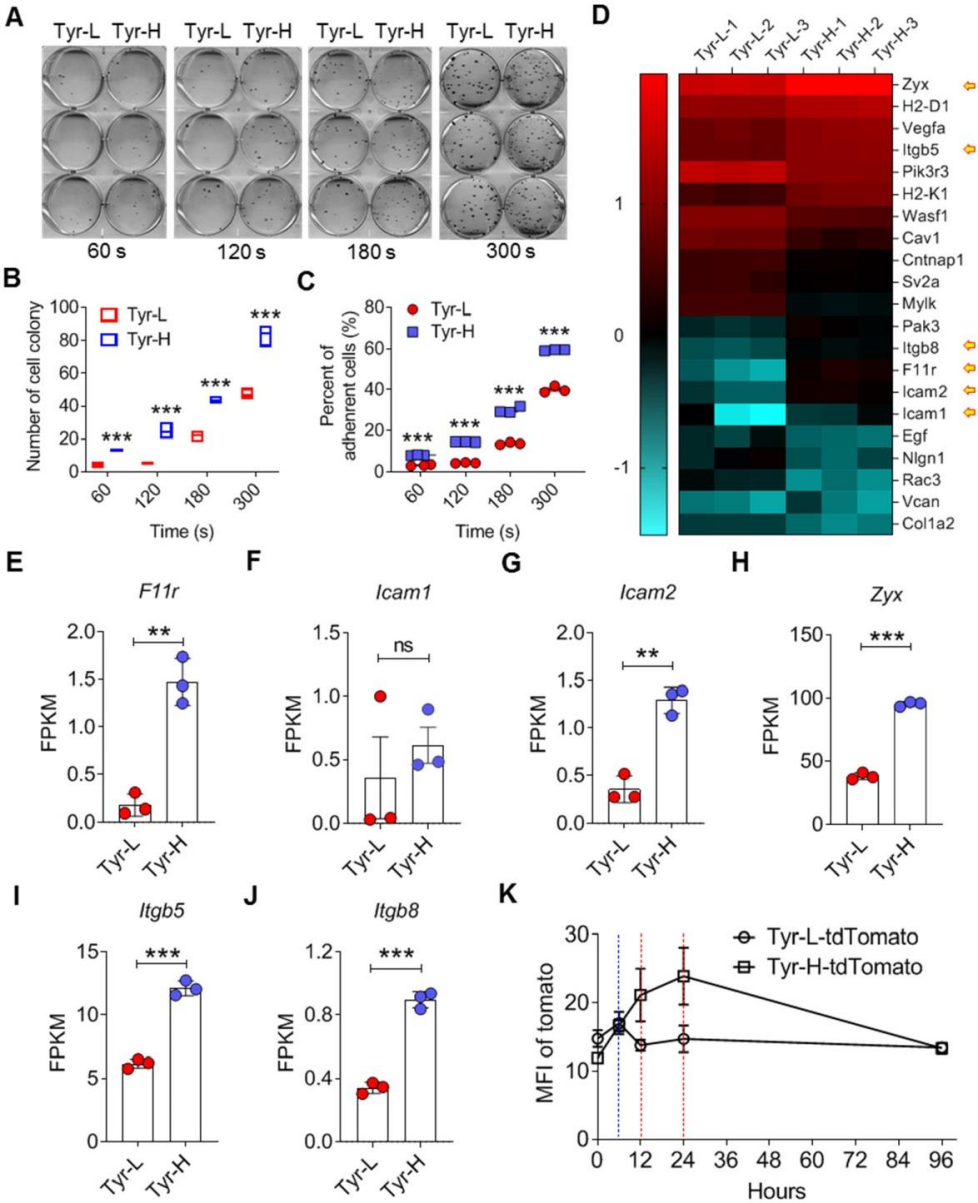
** Increased adhesiveness of Tyr-H cells. A-C.** Photos and counts of cell colony. Same number of Tyr-L cells and Tyr-H cells were seeded in the 6-well plate for 60 s, 120 s, 180 s or 300 s, and then discard the medium containing unattached cells. Cell colonies were counted after 10 days culture (A-B), or immediately counted and calculated the remained cells (C). **D.** Based on RNA sequencing, differentially expressed genes associated with cell adhesion between Tyr-L cells and Tyr-H cells were enriched by KEGG analysis and presented by heatmap. **E-J.** Based on RNA sequencing, expression of key genes associated with cell adhesion were showed. **K.** The ability of Tyr-L cells and Tyr-H cells to reside in the pulmonary tissues. Tyr-L cells and Tyr-H cells were labeled by tdTomato. All data given as mean ± SEM., n ≥ 3. **P < 0.01, ***P < 0.001, ns, not significant. All results shown are representative of three independent experiments.

We found that Tyr-H cells showed stronger adhesion ability (Figure 3A-C). RNA-seq results confirmed the upregulation of adhesion genes in Tyr-H cells, including *F11r*, *Icam1* and *Icam2* genes that are related to intercellular adhesion, and *Zyx*, *Itgb5* and *Itgb8* genes associated with the interactions between cells and the extracellular matrix (ECM) (Figure 3D-J). We further monitored the detainment of tdTomato expressing Tyr-H and Tyr-L cells in the lung when these cells were *i.v.* injected respectively. While similar frequencies of tdTomato expressing Tyr-H and Tyr-L cells were observed in the lungs within 6 h post cell injection (Figure 3K), Tyr-L cells almost disappeared at 12 h, in contrast to Tyr-H cells which remained high in number at 24 h post cell injection (Figure 3K). Thus, Tyr-H cells can easily inhabit the pulmonary tissue.

**Figure 4:**
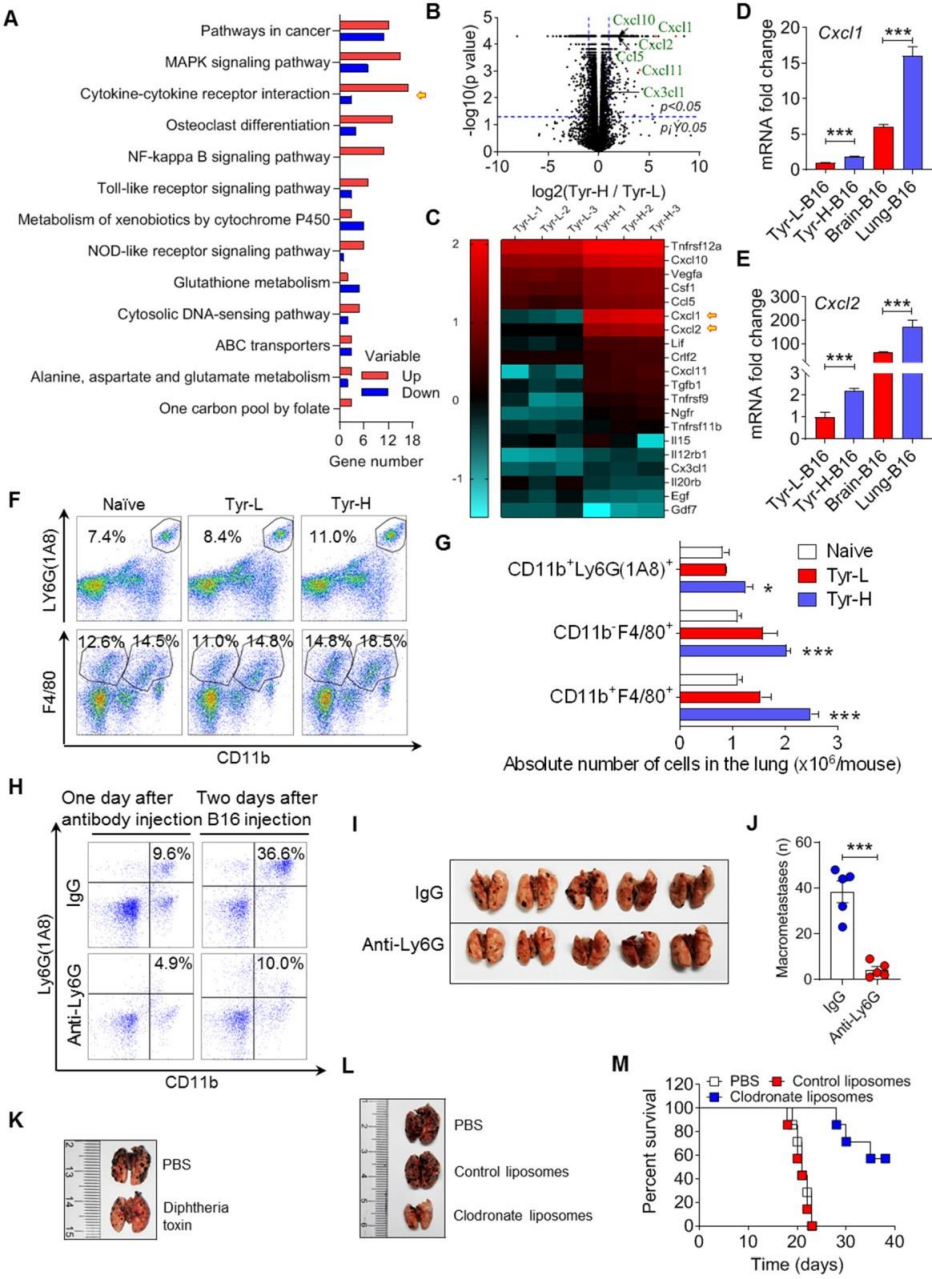
** Tyr-H cells recruit neutrophils and macrophages to construct pulmonary metastatic niche. A-C.** Gene expression profiles of Tyr-L cells and Tyr-H cells were analyzed by RNA sequencing. Differentially expressed genes participated signaling pathways were enriched by KEGG analysis (A). Volcano plot showing the differentially expressed genes (B). Cytokine-cytokine receptor interaction involved genes were represented by heatmap (C). **D-E.** Real-time quantitative PCR was used to analyze expression of* Cxcl1* gene and *Cxcl2* gene. Brain-B16 cells and Lung-B16 cells were isolated from experimental metastasis lesions respectively. **F-G.** Percentage of pulmonary tissues infiltrated CD11b^+^Ly6G (1A8)^+^ neutrophils, CD11b^-^F4/80^+^ alveolar macrophages (AMs) and CD11b^+^F4/80^+^ interstitial macrophages (IMs) gated in CD45^+^ cells (F). Absolute number of above cells in the lung was calculated (G). Lung tissues were collected on day 5 after the experimental pulmonary metastasis model was built. **H-J**, Neutrophils were depleted by Ly6G antibody *in vivo* before 48 h of B16 cell transplantation. Depletion efficiency and infiltration were detected by flow cytometry (H). Photos and counts of macrometastases in the lung from above mice (I-J).** K.** Representative photos of the metastasis lesions in the lung from experimental pulmonary metastasis mice. Normal B16 cells were intravenously injected into CD11b-DTR mice with or without diphtheria toxin treatment. **L-M.** Representative photos of the lung with metastasis lesions from experimental pulmonary metastasis model (L). Normal B16 cells were injected intravenously, with the treatment of PBS, control liposomes, and clodronate liposomes respectively. Percent survival was calculated (M). All data given as mean ± SEM., n ≥ 3. *P < 0.05, ***P < 0.001, ns, not significant. All results shown are representative of three independent experiments.

RNA-seq revealed that Tyr-H cells exhibited stronger pulmonary metastatic properties than those of Tyr-L cells (Figure 4A). Cytokine-cytokine receptor interaction genes were significantly enriched according to KEGG analysis. Tyr-H cells expressed higher levels of *Cxcl1*, *Cxcl2*, *Cxcl10*, *Cxcl11*, *Ccl5*, and *Cx3cl1*, which are responsible for myeloid cell infiltration, than Tyr-L cells (Figure 4B-C). Furthermore, B16 cells growing in the lungs expressed higher levels of *Cxcl1* and *Cxcl2* than those in the brain (Figure 4D-E), suggesting that Tyr-H cells and Tyr-L cells are distinct in their ability to construct metastatic immune microenvironment. Indeed, higher number of CD11b^+^Ly6G(1A8)^+^ neutrophils, CD11b^-^F4/80^+^ alveolar macrophages (AMs), CD11b^+^F4/80^+^ infiltrating macrophages were found in the lung in mice bearing Tyr-H cells (Figure 4F-G). Based on the GEPIA analysis 30, high level of *CXCL1*,* CXCL2* and *CXCR2* genes indeed often predicted the poor prognosis in melanoma patients (Figure S5A-F).

To verify the role of myeloid cells in regulating the colonization of Tyr-H cells in the lung, we used Ly6G neutralizing antibody to deplete neutrophils and found that the accumulation of neutrophils was limited and the pulmonary tumor burden of melanoma was decreased (Figure 4H-J). Next, we used diphtheria toxin to delete monocytes and macrophages in CD11b-DTR transgenic mice 31, 32, and found that the pulmonary colonization of Tyr-H cells was sharply suppressed (Figure 4K). Administration of clodronate liposomes that specifically deplete macrophages 33 also improved the survival of mice bearing Tyr-H melanoma (Figure 4L-M). Taken together, the colonization of Tyr-H cells in the lung relies on the infiltration of neutrophils and macrophages in the metastatic niche.

### Melanosome-dependent cell death supported training of pro-tumor macrophages

**Figure 5:**
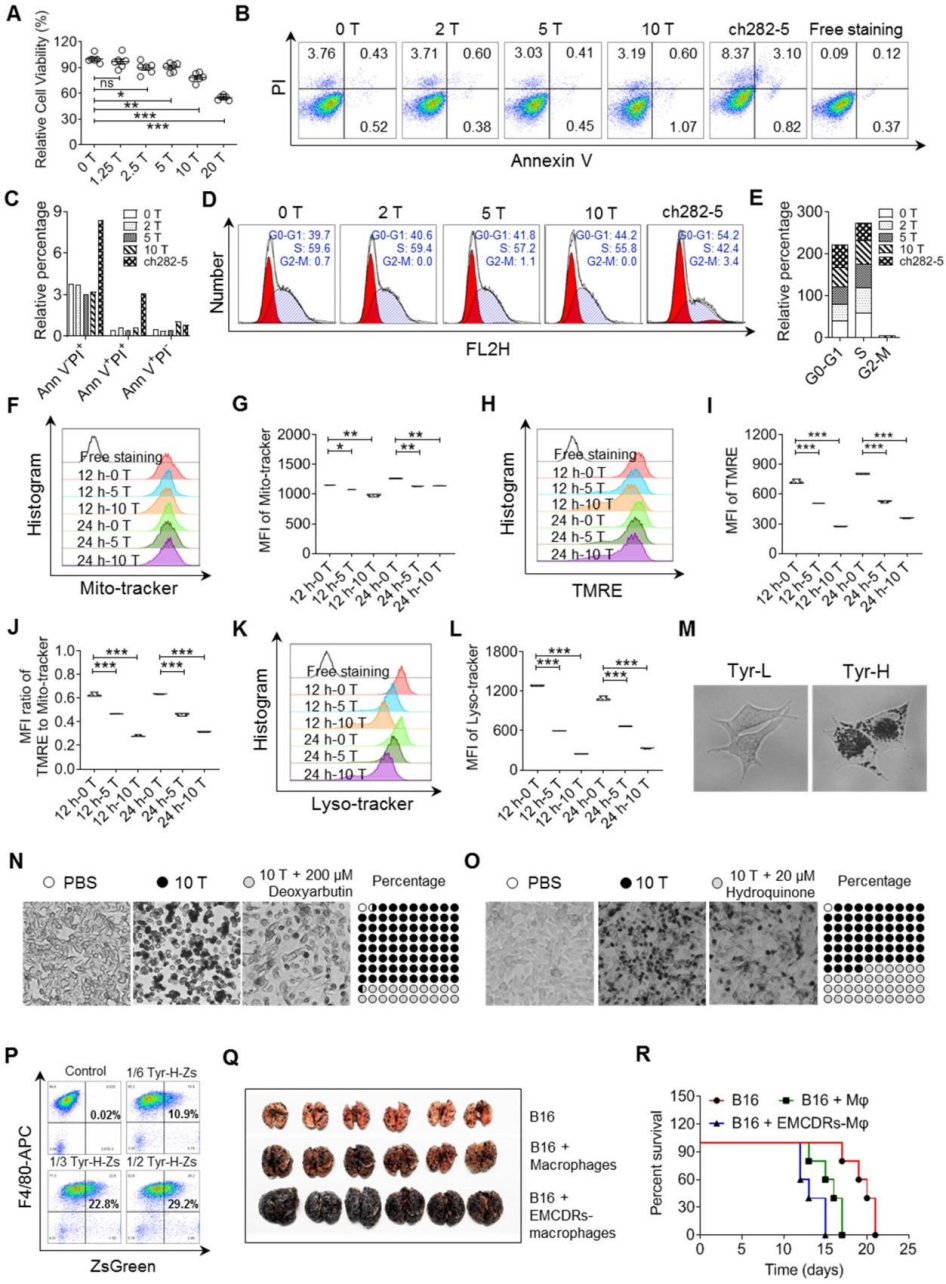
** Tyrosine-induced melanosome -dependent cell death supported the acquisition of tumor-promoting phenotype by macrophages. A.** Relative cell viability was evaluated by MTS assay. The same number of B16 cells were cultured in the conditional medium with different tyrosine addition for 24 h. **B-C.** Cell apoptosis was examined through Annexin V / PI staining. B16 cells were treated with different concentration of tyrosine or ch282-5 (Bcl-2 inhibitor, positive control). **D-E.** Cell cycle was analyzed by PI staining. **F-G.** Mitochondria was quantized by Mito-tracker staining. **H-J.** Mitochondrial membrane potential (MMP) dependent TMRE signaling was detected by flow cytometry (H-I). MMP was reflected by the MFI ratio of TMRE to Mito-tracker (J). **K-L.** Lysosome were quantized by Lyso-tracker staining. **M.** Representative photos of Tyr-L cells and Tyr-H cells with 10 T tyrosine for 24 h. **N-O.** Representative photos of B16 cells. B16 cells with 10 T tyrosine combining with or without deoxyarbutin or hydroquinone for 48 h. The percentage of pigment cells were calculated. **P.** Peritoneal macrophages were co-cultured with the EMCDRs (labeled with ZsGreen) in different ratios. Consuming efficiency were identified by flow cytometry. **Q-R.** Photos of the lung with metastasis lesions from experimental pulmonary metastasis model (Q). Normal B16 cells were injected intravenously, and mice were treated with PBS, macrophages, or EMCDRs-educated macrophages respectively. Percent survival was calculated (R). Mφ, macrophages; EMCDRs-Mφ, EMCDRs educated macrophages. All data given as mean ± SEM., n ≥ 3. *P < 0.05, **P < 0.01, ***P < 0.001, ns, not significant. All results shown are representative of three independent experiments.

Given that Tyr-H cells were more vulnerable to tyrosine exposure and that macrophages play a key role of in regulating their pulmonary colonization, we further questioned if macrophages contribute to the death and clearance of Tyr-H cells. We first explored how excess tyrosine induces B16 cell death. We found that initial addition of excess tyrosine in a concentration more than 2.5-fold of standard culture medium inhibited cell viability in a dose-dependent manner (Figure 5A), but without obvious apoptosis and cell cycle arrest (Figure 5B-E). Pan-caspase inhibitor (Z-VAD-FMK) and RIP1 inhibitor (necrostatin-1) did not reverse the impairment of Tyr-H cell viability upon exposure to excess tyrosine (Figure S6A). These data suggest that impairment of Tyr-H cell viability induced by excess tyrosine is not related to classical apoptosis and necrosis. Reactive oxygen species (ROS) are always entangled in chemoresistance or death of tumor cells 34, however, the level of mitochondrial ROS was not enhanced in the presence of excess tyrosine, as shown by Mitosox-Red staining (Figure S6B-C).

We found that excess tyrosine slightly decreased the quantity of mitochondria and sharply impaired mitochondria membrane potential (MMP, figure 5F-J). Therefore, excess tyrosine might suppress cell viability through limiting mitochondria respiration. Previous studies have demonstrated that mitochondrion-lysosome cross-talk always affects proton rechanneling-dependent cell metabolism, cell viability and cell death 35, 36. Consistently, excess tyrosine significantly reduced the abundance of lysosomes (Figure 5K-L). We wondered if the excess melanosome assembly mobilized by tyrosine might have plundered the inner membrane resources and thus limit lysosome biogenesis. Upon treatment with 10-fold higher than standard tyrosine, a large accumulation of melanosomes occurred in Tyr-H cells, but not in Tyr-L cells (Figure 5M). When tyrosinase inhibitors, deoxyarbutin and hydroquinone, were respectively added into the culture medium 37, 38, uncontrolled melanosome synthesis and cell death were suppressed (Figure 5N-O). Thus, exposure to superabundant tyrosine induces an excess of melanosome formation in Tyr-H cells and leads to their death.

We further demonstrated that macrophages acquired a pro-tumor phenotype through engulfing the remains of excess melanosome induced cell death (EMCDRs), and these macrophages promoted the colonization of B16 cells to the lung (Figure 5P-R). In addition, these Tyr-L and B78H1 cells that hardly colonize in the lung can grow when i.v. injected with B16 EMCDRs (Figure S3G and S6D-E). Taken together, Tyr-H cells rely on their own sacrifice that confers macrophages tumor-promoting properties and consequently enables lung colonization.

### Tyr-L cells enhance PD-L1 expression in the cerebral melanoma infiltrated macrophages

**Figure 6:**
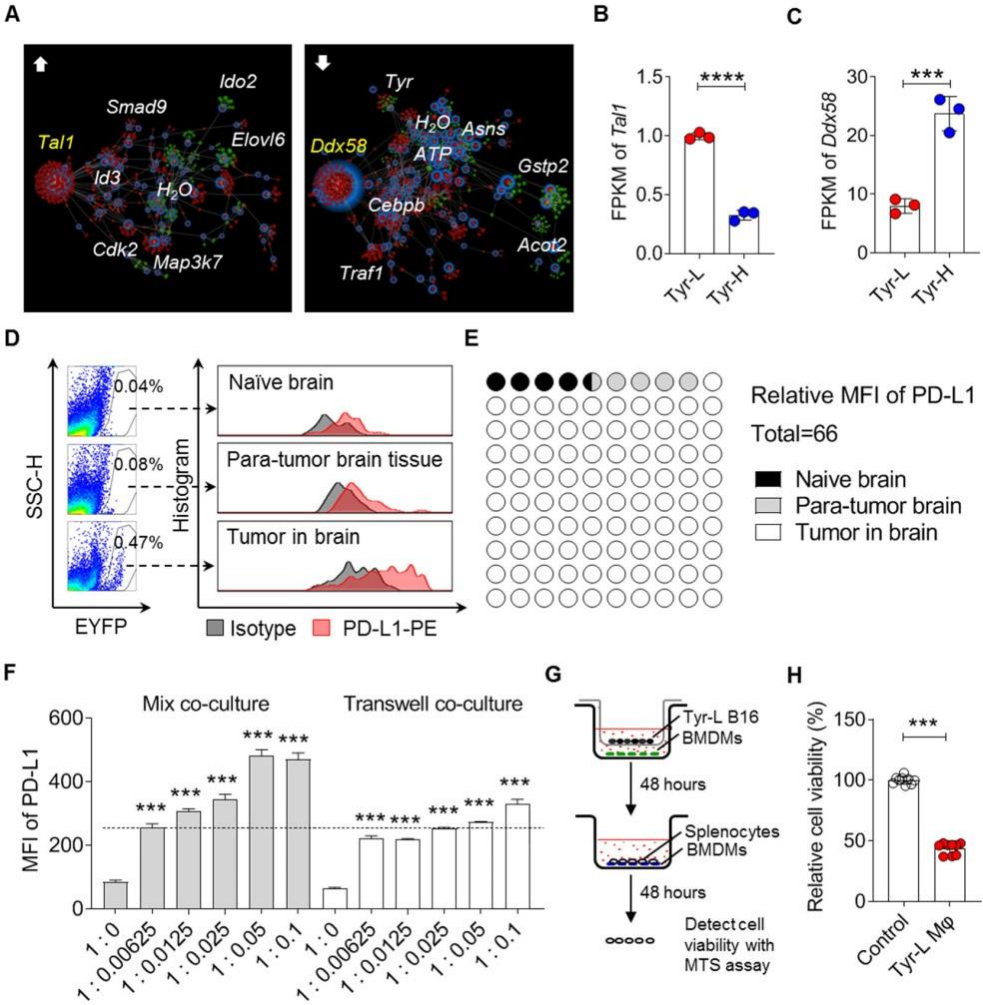
** Tyr-L cells enhance PD-L1 expression in the cerebral melanoma infiltrated macrophages. A.** Comparing to Tyr-H cells, interaction of high expression of (left) and low expression of (right) genes (red nodes) and their associated metabolites (green nodes) in Tyr-L cells were analyzed by the web of OmicsNet. **B-C**. mRNA level of core genes enriched in the above analysis. **D-E.** B16 melanoma cells were transplanted into the mice by stereotactic injection. PD-L1 expression of macrophages in the naïve mouse brain, para-tumor tissue and tumor were analyzed by flow cytometry (D), and the MFI of PD-L1 was calculated (E). **F.** Bone marrow derived macrophages (BMDMs) were co-cultured with Tyr-L cells in the mixture co-culture system or transwell co-culture system for 48 h. PD-L1 expression were detected by flow cytometry. **G-H.** Splenocytes were co-cultured with Tyr-L cells educated BMDMs. Relative splenocyte viability were measured by MTS assay. All data given as mean ± SEM., n ≥ 3. ***P < 0.001, ****P < 0.0001, ns, not significant. All results shown are representative of three independent experiments.

Malignant melanoma is the third most common type of tumor that results in brain metastases 39. We employed OmicsNet to analyze the interactions of up- or down-regulated genes and their associated metabolites in Tyr-L cells, and found that upregulated *Tal1* and downregulated *Ddx58* were core genes (Figure 6A-C). It has been reported that upregulation of Tal1 promotes glioma progression, possibly by augmenting PD-L1 expression 40, 41. Whether PD-L1 expression involved in Tyr-L cell progression in brain needs to be studied further. Additionally, it has been found that* Ddx58* coded RIG-I is critical for responsiveness to anti-CTLA4 therapy in tumor 42. Expression of *Ddx58* significantly decreased in Tyr-L cells, indicating that Tyr-L cells may not be sensitive to the anti-CTLA4 therapy. PD1 antibody, as an immune checkpoint inhibitor, has been firstly utilized in the treatment of cerebral metastatic melanoma 39. Considering that Tyr-L cells can rapidly proliferate in the brain, we wondered if Tyr-L cells enhanced the PD-L1 expression in the cell components of tumor niche. As expected, lyz2-EYFP tracing myeloid cells showed higher level of PD-L1 expression than that in the myeloid cells derived from naïve mouse brain tissue and para-tumor brain tissue (Figure 6D-E).

Employing the mixture co-culture system and transwell co-culture system, we confirmed that Tyr-L cells promoted PD-L1 expression in macrophages (Figure 6F). The PD-L1 upregulation might depend on both cell-cell interaction and paracrine factors. Macrophages co-cultured with Tyr-L cells exhibited enhanced ability to suppress the viability of splenocytes (Figure 6G-H). Therefore, the colonization of Tyr-L cells in the brain could be attributed to the induction of PD-L1 expression in infiltrating macrophages to form immunosuppressive microenvironment.

### Targeting tyrosine utilization synergized immune checkpoint blockade therapy

**Figure 7:**
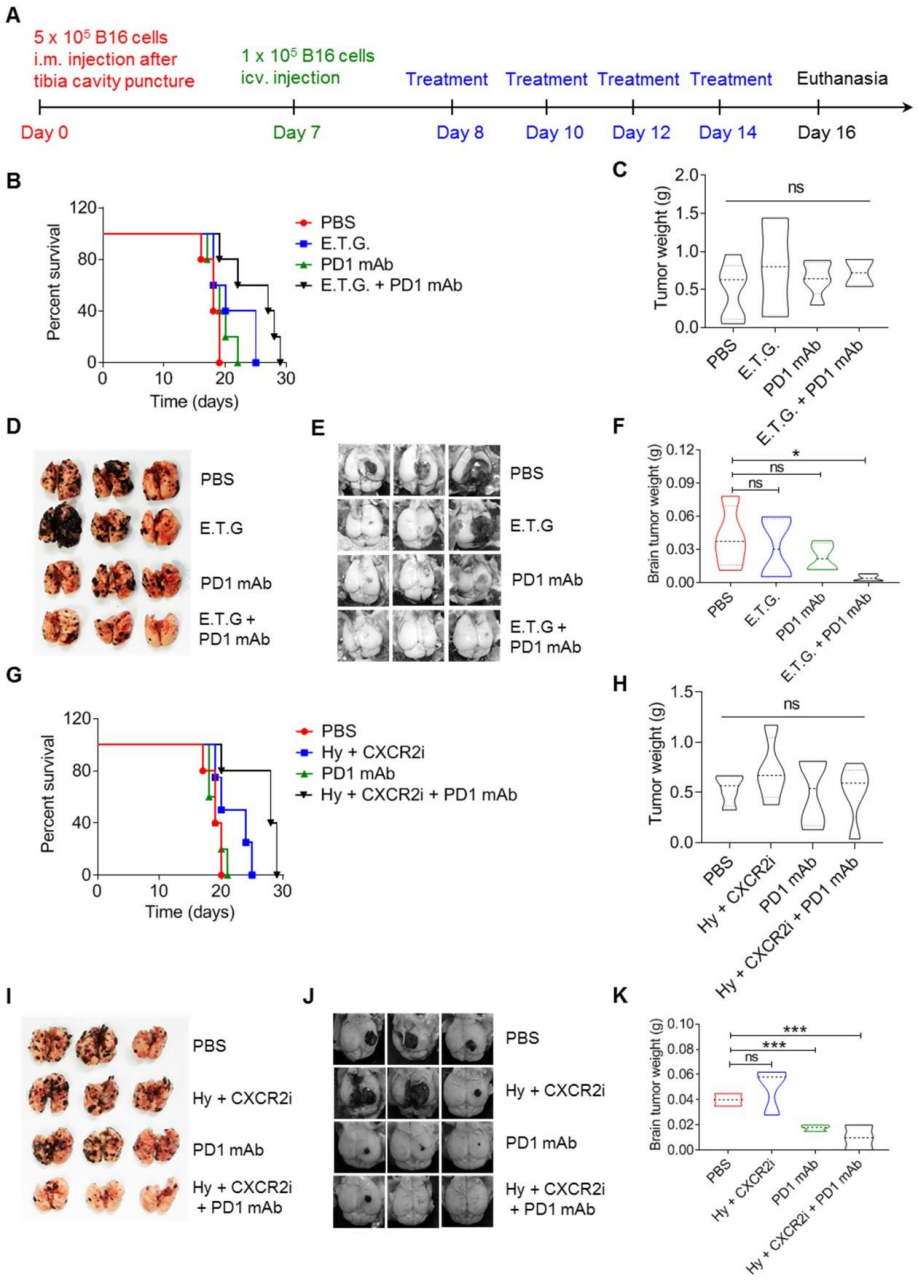
**Targeting tyrosine utilization augments the therapeutic effects of PD1 monoclonal antibody. A.** The schedule showing composite melanoma model and treatment. **B-F.** Composite melanoma mice were treated with E.T.G. (2-Ethoxybenzamide, tyrosine, plus GSK343), PD1 monoclonal antibody (mAb), and E.T.G. plus PD1 mAb, PBS treatment as a control. Percent survival (B), in situ tumor mass (C), pulmonary macrometastases (D), cerebral colonization (E) and cerebral tumor weight (F) were observed. **G-K.** Composite melanoma mice were treated with hydroquinone plus CXCR2 inhibitor SB225002 (Hy + CXCR2i), PD1 mAb, and Hy + CXCR2i plus PD1 mAb, PBS treatment as a control. The indicators as panel B-F were observed. All data given as mean ± SEM., n ≥ 3. *P < 0.05, ***P < 0.001, ns, not significant. All results shown are representative of three independent experiments.

Given that the presence of extracranial tumor is necessary for the efficacy of immunotherapy against distant brain metastatic tumor 26, a modified composite model of melanoma with primary tumor inoculation in the muscle and distant colonization in the lung and brain was used to explore anti-tumor strategy (Figure 1H and 7A). Considering the heterogeneity of B16 cells in tyrosinase-controlled utilization of tyrosine, we compared the expression of amino acid metabolism associated genes and next sought to develop strategy to combat their organotropic colonization (Figure S7A). We first used 2-Ethoxybenzamide (2-ETZ), an effective tyrosinase agonist 43 to treat B16 cells and found that the synthesis of melanin in both Tyr-H and Tyr-L cells were enhanced (Figure S7B). Different from high expression of adhesion genes and chemotaxis genes in Tyr-H cells under the general tyrosine concentration, excessive mobilization tyrosinase through addition of 2-ETZ and tyrosine significantly decreased the expression of genes related to cell adhesion and chemotaxis, such as* F11r, Itgb5*, *Icam1*, *Icam2*, *Cxcl1*, *Cxcl2*, *Cxcl11*, and *Cx3cl1* in either normal B16, Tyr-L, or Tyr-H cells (Figure S8A-D). These data suggest that excessive activation of tyrosinase may suppress the adhesive ability of B16 cells and their function in recruitment of myeloid cells. Macrophages are important in promoting the colonization of B16 cells in the lung via engulfing EMCDRs of B16 cells (Figure 5P-R and figure S6D-E). GSK343 and GSK503 as the selective EZH2 inhibitor have been shown to have therapeutic effects on pediatric glioma and melanoma 44-46. Furthermore, while GSK343 inhibits phagocytosis of *E. coli* by macrophages 47, we also found that GSK343 can also impair the phagocytosis of EMCDRs by macrophages *in vitro* (Figure S7C-E). Therefore, GSK343 was used to suppress EMCDR-mediated tumor-promoting polarization of macrophages in mice bearing B16 melanoma. While no significant therapeutic effect can be observed in E.T.G. (2-ETZ + tyrosine + GSK343) treated group, its combination with PD1 monoclonal antibody (mAb) significantly prolonged the survival of mice bearing B16 melanoma and decreased the number of nodules in the lung and brain (Figure 7B-F).

It was reported that hydroquinone, as a tyrosinase inhibitor, prolongs the life of the melanoma bearing mice 48, 49. Additionally, it has been found that limiting neutrophils infiltration into the pancreatic tumor and lung cancer by CXCR2 inhibitor can promote tumor infiltration of CD8^+^ T cells and enhance their anti-tumor abilities 50, 51. As shown in Figure 1 and Figure 2, restriction of melanin production of B16 cells abolished their colonization in the lung, it, however, enhanced their potential to form brain metastasis. Also, Tyr-H cells expressed high levels of CXCR2 ligands for recruiting neutrophils and macrophages (Figure 4). Taken together, we proposed a combination strategy of using inhibitors for melanin production and CXCR2. More significant therapeutic effects were observed in B16 melanoma bearing mice treated with three-drug combination of hydroquinone (Hy), CXCR2 inhibitor (SB225002, CXCR2i) 49, 50, 52 and PD1 mAb, as manifested by the prolonged survival time and reduced tumor burden in the lung and brain (Figure 7G-K). Thus, targeting the heterogeneity of B16 cells in tyrosine utilization can shape tumor microenvironment and enhance the therapeutic effect of PD1 mAb. Mechanistically, hydroquinone and SB225002 abolished the formation of pro-tumor immune microenvironment in the lung, and PD1 mAb inhibited the tumor growth in the brain through destroying PD1-PD-L1 immune checkpoint.

## Discussion

Melanoma harbors different mutations contributing to the intratumor heterogeneity, and the subclones selectively metastasize to distinct organs 1.Culture conditions* in vitro* often select for sub-populations with particular gene expression patterns from primary cancer cells, which in turn affects subsequent experiments and interpretations 53, 54. For example, toxin lectin enriched 70-W variant from MeWo melanoma cells showed more strong metastatic ability to skin, brain, bone marrow, ovary, mesentery and muscle, while MeWo cells only metastasized to the lung 55. We found that lung colonizing B16 melanoma cells were apt to utilize tyrosine for melanin production when they were cultured *in vitro*. Excessive melanin production caused some of them to die, and the debris-engulfing macrophages thus acquired tumor-promoting property. In contrast, Tyr-L cells, which have low utilization of tyrosine for melanin production, are poised to colonize the brain. Our findings are in line with the notions that melanogenesis and melanin pigment affect the behavior of malignant melanocytes 56, and targeting melanogenesis may represent a potential adjuvant treatment strategy for melanotic melanoma 57. Heterogeneity of melanoma cells in tyrosine utilization thus opens a new venue for combating pulmonary and cerebral metastasis of melanoma.

The roles of amino acids in regulating the behavior of cancer cells and immunocytes are well recognized. For example, tyrosine supported melanogenesis leads to HIF-1α stabilization and HIF-dependent phenotypes in melanoma 58, melanotic melanoma cells secreted L-DOPA inhibits induced proliferation of murine and human lymphocytes 59, 60. Nevertheless, how melanoma cells with special tyrosine metabolic characteristics drive the formation of organotropic colonization microenvironment is still poorly understood. We noticed a significant variation of tyrosine levels among multiple organs during B16 melanoma progression. Although tyrosine was not shown to attract B16 cell migration in* in vitro* experiment, similar content of tyrosine in the small size tumor, pulmonary tissue and cerebral tissue still implicated tyrosine metabolism in the organotropic metastasis of melanoma. Indeed, cell subpopulations isolated from multiple organ metastasis responded similarly to tyrosine, except that those colonizing the lung produced more melanin under tyrosine treatment, which once again confirms that Tyr-H cells have an advantage in lung colonization. We found that Tyr-H cells had stronger adhesion ability and attracted more neutrophils and macrophages into pulmonary tissue. However, how tyrosinase becomes adequately activated to drive the expression of genes associated with adhesion and chemotaxis remains to be further investigated.

Patient-derived pigmented melanoma cells (Figure 1A, 238818) and Tyr-H cells were all extremely efficient in converting tyrosine into melanin. However, most of these cells inevitably died. These dead cells conferred macrophages with immunosuppressive ability. Interestingly, the tyrosine sensitive melanoma cells did not undergo classical apoptosis or necrosis. Excess tyrosine treatment sharply reduced mitochondria membrane potential (MMP). Decreased MMP is generally associated with mitochondrial damage and apoptosis 36, however, no obvious Annexin V / PI staining can be detected in the tyrosine treated B16 cells. Mitochondria quality and quantity is associated with lysosome-dependent mitophagy 35, 36. Surprisingly, tyrosine treatment reduced the abundance of lysosome to one-fifth of the original level. Imbalanced distribution of inner membrane resources may disrupt the homeostasis of membrane-bound organelles. The excessive melanosome generation may have impaired the assembly of lysosome. Importantly, inhibiting melanin synthesis limited pigment melanosome accumulation in the B16 cells and deceased cell death to some extent. Therefore, tyrosine caused B16 cell death via excessive melanin synthesis and melanosome accumulation, which may have been exacerbated by the decreased MMP and lysosomal quantity.

We explored some strategies for suppressing melanoma cell metastasis to the lung and brain through modulating tyrosinase activity. In one strategy, tyrosinase activity and melanin synthesis were elevated by tyrosinase agonist 2-ETZ and melanin substrate tyrosine, which will enhance cell death through excessive melanosome accumulation. When combined with GSK343 to inhibit the phagocytosis of dead cells, the macrophages could no longer acquire their immunosuppressive property. In another strategy, we inhibited tyrosinase using hydroquinone to promote the survival of Tyr-H cells. Considering that Tyr-H cells express high levels of chemokines, CXCR2 inhibitor SB225002 could be used to block the recruitment of neutrophils and macrophages. The two strategies synergized with PD1 antibody to exert more potent therapeutic effects. Although the therapeutic effects of combination strategies were demonstrated in the B16 composite tumor model, the actual tumors in patients may not completely correspond to the heterogeneity of B16. A more appropriate combination strategy may require individual adjustments.

## Materials and Methods

### Clinical samples

Patient derived 255776 melanoma cells and 238818 melanoma cells were provided by Fujian Provincial Cancer Hospital. These cells were cultured in DMEM medium (with addition of 10% fetal bovine serum, 1% penicillin & streptomycin and 1% glutamax) in a 5% CO_2_ humidified atmosphere at 37 °C. All procedures were conducted in accordance with the Declaration of Helsinki and with approval from the ethics committee of Fujian Medical University Cancer Hospital (Ethics Number: 2019-083). Written informed consent was obtained from all participants.

### Mice

C57BL/6 mice were purchased from Shanghai Laboratory Animal Center of the Chinese Academy of Science (Shanghai, China). EYFP*^fl/fl^* mice were purchased from Jackson Laboratories (Bar Harbor, ME). Lyz2*^Cre^* and CD11b-DTR mice were provided by Dr. Honglin Wang, Shanghai Jiao Tong University School of Medicine. All mice were C57BL/6 background. EYFP*^fl/fl^* mice were crossed with Lyz2*^Cre^* mice to generate mice with EYFP-specific expression in myeloid cells only. Mice used in this study were 9- to 12-week old males. Mice were maintained in a specific pathogen-free facility of the Shanghai Institute of Nutrition and Health of the Chinese Academy of Sciences and were used in accordance with guidelines of the Institutional Animal Care and Use Committee.

### Subclone selection of Tyr-H cells and Tyr-L cells

*Monoclonal assay.* Cell suspensions were added into the 10 cm dish at a density of 1,000 cells per 10 mL culture medium. Seven days later, monoclones can be collected and expended for further experiments.

### *In vitro* and *in vivo* enrichment of Tyr-L cells

*In vitro enrichment.* B16 cells were cultured for more than 20 passages in the conditional medium with 2-fold, 5-fold and 10-fold higher than standard concentration of tyrosine (2 T, 5 T, and 10 T, standard concentration of tyrosine in culture medium is 103.79 mg/L in the form of L-Tyrosine disodium salt dihydrate). The pigment of tyrosine treated melanoma cells turn from light to dark, and then to light again.

*In vivo enrichment.* B16 cells were transplanted into mice by *s.c*. injection. When the diameter of tumors arrived at 5 mm, tyrosine (104 μg/μL, 50μL) was injected into the tumor every the other day for 7 times. Tumor tissues were digested by the type I collagenase (Sigma Aldrich, 2 mg/mL, add 10% fetal bovine serum) into single cells. Then the primary cells were cultured in DMEM medium (with addition of 10% fetal bovine serum, 1% penicillin & streptomycin and 1% glutamax) in a 5% CO_2_ humidified atmosphere at 37 °C.

### Tyrosinase activity assay

Tyrosinase activity was determined with Tyrosinase Activity Assay Kit (Abcam, cat. no. ab252899), following the recommended protocols. Tyr-L B16 cells and Tyr-H B16 cells were cultured in DMEM medium (with addition of 10% fetal bovine serum, 1% penicillin & streptomycin and 1% glutamax) in a 5% CO_2_ humidified atmosphere at 37 °C. Parental B16 melanoma cells were cultured as above with or without 2-ETZ or L-tyrosine addition for another 24 h before determination of protein expression and tyrosinase activity.

### Cell viability assay

Three thousand or 5,000 cells were treated with tyrosine or other combinations for different time in 96-well plates in a final volume of 100 μL and viability was assessed by MTS assay (CellTiter 96 AQueous One Solution Cell Proliferation Assay, Promega).

### Whole body metastasis model of melanoma

B16 cells were injected into the left cardiac ventricle (2 × 10^5^ cells in 100 μL PBS) with 27-gauge needle under the anesthesia. Mice were euthanized 30 days after modeling, and tissues were taken to separate cells.

### Composite melanoma models and the treatment schedules

*Composite model with tumor in muscle and pulmonary metastasis focuses.* The tibia cavity was perforated with 26-gauge needle, and then 5 × 10^5^ B16 cells in 50 μL PBS were injected into the muscle between tibia and fibula with 29-gauge needle. Fifteen to thirty days later, mice were euthanized and collected the melanoma tumor from the muscle tissues and lung tissues.

*Experimental pulmonary metastasis model.* B16 cells (5 × 10^5^ B16 cells in 200 μL PBS) were transplanted into mice through *i.v.* injection. Fifteen days later, mice were euthanized and collected the pulmonary tissues.

*Experimental cerebral metastasis model.* B16 cells (1 × 10^5^ B16 cells in 5 μL PBS) were stereotactically injected into the brain (2.5 mm anterior from bregma, 2 mm right from the midline, 2 mm deep). Seven to fourteen days later, mice were euthanized and collected the cerebral melanoma tissues.

*Improved composite model* was built through superimposing the experimental cerebral metastasis model on the basis of above composite model after 7 days.

*Treatment.* The treatment schedule of the improved composite model mice was described in Fig. 7A. The dosages were modified referring to literatures. PD1 monoclonal antibody (Bio X Cell Cat# BP0146, RRID: AB_2894808) and IgG2a isotype control (Bio X Cell Cat# BP0089, RRID: AB_2894744) were* i.v.* injected with the dosage of 200 μg per mouse 26. The following treatments are administered subcutaneously every the other day: 2-ETZ (Sigma Aldrich, E4402) at the dosage of 40 mg/kg. Tyrosine (Sigma Aldrich, T8566) at the dosage of 10 mg/kg. GSK343 (Selleck, S7164) at the dosage of 10 mg/kg. Hydroquinone (Selleck, S4580) at the dosage of 50 mg/kg. SB225002 (Selleck, S7651) at the dosage of 5 mg/kg.

### Fluorescence labeled B16 cells

Tyr-L cells and Tyr-H cells were transfected with the retroviruses carried ZsGreen gene or tdTomato gene respectively. Then the sensitivities of tyrosine utilization were evaluated before further experiment.

### *In vitro* and *in vivo* evaluation of adhesion ability

*In vitro evaluation.* Same number of Tyr-L cells and Tyr-H cells were respectively planted in the 6-well plate for 60 s, 120 s, 180 s or 300 s, and then discard the medium containing unattached cells. Then the remaining cells were calculated immediately, or cultured for 10 days and cell colonies were stained with 0.1% crystal violet.

*In vivo evaluation.* Same number of Tyr-L-tdTomato cells and Tyr-H-tdTomato cells were respectively transplanted into mice by* i.v.* injection. TdTomato signaling was evaluated with flow cytometry at 0 h, 6 h, 12 h, 24 h, and 96 h.

### Co-culture experiment

*Mixture co-culture.* Bone marrow derived macrophages (BMDMs) were cultured in the RIPM-1640 medium with the 20 ng/mL macrophage colony stimulating factor (M-CSF, R&D Systems), 10% fetal bovine serum, 1% penicillin & streptomycin and in a 5% CO_2_ humidified atmosphere at 37 °C for 6 days. Then B16 cells were added into the dish in different ratio and co-cultured for 48 h.

*Transwell co-culture.* Above BMDMs were seeded in the bottom chamber of transwell (6 wells, 0.4 μm pore size with polycarbonate membrane; Corning Costar), and B16 cells were added into the top chambers in different ratio and co-cultured for 48 h.

### Invasion and migration assay

Invasion and migration were performed as our previously reported 36. For invasion assay, the transwell system (24 wells, 8 mm pore size with polycarbonate membrane; Corning Costar) was coated with 3 mg/mL matrigel (BD Biosciences). Three h later, 1 × 10^5^ Tyr-L cells or Tyr-H cells in 500 μL tyrosine-free DMEM medium were seeded into the top chambers and 500 μL DMEM medium containing 1T standard tyrosine was added into the bottom chamber. Forty-eight h later, cells that did not invade through the pores of the transwell inserts were removed with a cotton swab and the inserts were fixed in cold methanol for 10 min and then stained with 0.1% crystal violet. For migration assay, the experiments were performed as above except that cells were plated on top of uncoated (matrigel-free) inserts. The migrated or invaded cells were photographed under a light microscope.

### Transcriptome sequencing

The transcriptome sequencing experiments were performed by Vazyme Biotech (Nanjing, China). Total RNA of Tyr-L cells and Tyr-H cells were extracted with TRIzol reagent (Invitrogen). The transcriptome library was generated utilizing VAHTS mRNA-seq v2 Li-brary Prep Kit for Illumina (Vazyme Biotech) following the manufacturer's recommendations. The index-coded samples were clustered through VAHTS RNA Adapters set1/set2 for Illumina (Vazyme Biotech). And then, the libraries were sequenced on Illumina Hiseq X Ten platform utilizing paired-end module (2 × 150 bp). *Q value ≤ 0.05* and *fold-change ≥ 2* between 2 samples were considered the genes with different expression level.

### Immunofluorescence analysis

*Cell membrane surface proteins* were detected after blocking non-specific staining by incubation with anti-CD16/32 (eBioscience). Cells were then stained with antibodies for 30 min at room temperature. The antibodies specific for surface proteins were CD45 (Thermo Fisher Scientific Cat# 35-0451-80, RRID: AB_11218508), CD11b (Thermo Fisher Scientific Cat# 17-0112-82, RRID: AB_469343), F4/80 (Thermo Fisher Scientific Cat# 11-4801-85, RRID: AB_2637192), Ly6G (1A8) (BioLegend Cat# 127616, RRID: AB_1877271), Ly6C (Thermo Fisher Scientific Cat# 12-5932-82, RRID: AB_10804510), and PD-L1 (Thermo Fisher Scientific Cat# 12-5982-83, RRID: AB_466090).

*Nuclear protein detection.* Melanoma cells were subjected to the Foxp3 Transcription Factor Staining Buffer (eBioscience). Cells were then stained with Ki67 antibody (BioLegend Cat# 652410, RRID: AB_2562141) at room temperature for 30 min.

*Cell apoptosis and cell cycle analysis* was performed as our previously reported 36, depending on the Annexin V / Propidium Iodide (eBioscience) staining and Propidium Iodide (Sigma Aldrich, 5 mg/mL, with RNAase addition) staining.

*Mitochondrial membrane potential (MMP).* Cells were stained with TMRE and mito-tracker at 37 ºC for 30 min. The MFI ratios of TMRE to mito-tracker were calculated.

*Lysosome* were detected by staining cells with Lyso-tracker Red DND-99 (Invitrogen) at 37 ºC for 30 min.

*Mitochondrial ROS* were measured by staining cells with Mitosox-Red (Invitrogen) at 37 ºC for 15 min.

### Measurement of tyrosine concentration

Mouse tissue samples were weighed and broken in ultrapure water with a tissue homogenizer. Then the tyrosine concentration of tissue soluble samples or culture medium were measured by Tyrosine Colorimetric Assay Kit (Sigma Aldrich).

### Measurement of melanin

Cell samples were boiled in the 2 mM NaOH solution at 100 ºC for 30 min, and the absorbance of melanin at 405 nm was measured. Melanin concentration was calculated through comparing with the standard curve.

### Real-time quantitative PCR (RT-PCR)

Transcriptional expression determination was performed as our previously reported 35. Total RNA was extracted with the RNAprep pure Cell/Bacteria Kit (Tiangen Biotech) and reverse-transcribed using the PrimeScript RT Master Mix (Takara). The FastStart universal SYBR Green master (Roche) was added to each PCR reaction along with cDNA and specific primers to a total volume of 10 μL. Real-time qPCR was performed on an ABI Prism 7900HT (Applied Biosystems). Primers specific for *Cxcl1*: F-ACTGCACCCAAACCGAAGTC, and R-TGGGGACACCTTTTAGCATCTT; Primers specific for *Cxcl2*: F-CCAACCACCAGGCTACAGG, and R-GCGTCACACTCAAGCTCTG.

### Statistical Analysis

Data are provided as mean or mean ± SEM. Populations were compared using *t-test*, two-tailed unpaired Student's, with a 95% confidence interval under the untested assumption of normality. For all tests, *p*-values are indicated as: *P < 0.05, **P < 0.01, ***P < 0.001, ****P < 0.0001, and ns means not significant. *P < 0.05* was considered statistically significant.

## Supplementary Material

Supplementary figures.

## Abbreviations

L-DOPA: L-dihydroxyphenylalanine
Tyr-H cells: B16 melanoma cells with high activity of tyrosinase and sensitivity of tyrosine utilization for melanin synthesis
Tyr-L cells: B16 melanoma cells with low activity of tyrosinase and insensitivity of tyrosine utilization for melanin synthesis
2 T, 5 T, and 10 T: 2-fold, 5-fold and 10-fold higher than standard concentration of tyrosine
EMCDRs: remains of excess melanosome induced cell death
PD1 mAb: PD1 monoclonal antibody
ECM: extracellular matrix
AMs: alveolar macrophages
ROS: Reactive oxygen species
2-ETZ: 2-Ethoxybenzamide
E.T.G.: 2-Ethoxybenzamide + tyrosine + GSK343
CXCR2i: CXCR2 inhibitor, SB225002
MMP: mitochondria membrane potential
