# Supplementary material for: Heterogeneity of tyrosine-based melanin anabolism regulates pulmonary and cerebral organotropic colonization microenvironment of melanoma cells
Source: Theranostics. 2022 Jan 31;12(5):2063–79. doi: 10.7150/thno.69198 (PMC8899568; doi:10.7150/thno.69198)
Supplement: Supplementary file 1 — Supplementary figures. [file thnov12p2063s1.pdf]

Supplementary Materials

Heterogeneity of tyrosine-based melanin anabolism regulates pulmonary and cerebral organotropic colonization microenvironment of melanoma cells

Xuefeng Wang[1,2], Yu Chen[3], Bin Lan[4], Yu Wang[2], Wansong Lin[3], Xu Jiang[2], Jiayin Ye[2], Bingxue Shang[1], Chao Feng[1,5], Jun Liu[3], Jingjie Zhai[2], Muhan Xu[2], Qing Li[2], Liangyu Lin[2], Mingyuan Hu[2], Fanjun Zheng[2], Ling Chen[3], Changshun Shao[1,*], Ying Wang[2,*], and Yufang Shi[1,2,5,*]

[1]The Third Affiliated Hospital of Soochow University and State Key Laboratory of Radiation Medicine and Protection, Institutes for Translational Medicine, Soochow University, 199 Renai Road, Suzhou, Jiangsu 215123, China.

[2]CAS Key Laboratory of Tissue Microenvironment and Tumor, Shanghai Institute of Nutrition and Health, University of Chinese Academy of Sciences, Chinese Academy of Sciences, 320 Yueyang Road, Shanghai 200031, China.

[3]Fujian Provincial Key Laboratory of Translational Cancer Medicine, Cancer Bio-immunotherapy Center, Fujian Medical University Cancer Hospital, 420 Fuma Road, Fuzhou 350014, China.

[4]Shanghai Jiao Tong University School of Medicine, Shanghai Center for Systems Biomedicine Research, Shanghai Jiao Tong University, 800 Dongchuan Road, Shanghai 200240, China.

[5]Department of Experimental Medicine, TOR, University of Rome Tor Vergata, Rome 00133, Italy.

***Correspondence:**

yfshi@suda.edu.cn (Y.S.), yingwang@sibs.ac.cn (Y.W.), shaoc@suda.edu.cn (C.S.).

Supplementary Materials

Figure. S1. Tyrosine concentration in multiple organs have dynamic changes following tumor progression.
Figure. S2. Expression of melanogenesis related proteins and tyrosinase activity.
Figure. S3. Tyrosine did not provide stronger chemotaxis for Tyr-H cells than Tyr-L cells.
Figure. S4. Excess tyrosine enriches Tyr-L B16 cells.
Figure. S5. High level expression of CXCL1, CXCL2 and CXCR2 predicts poor prognosis of melanoma.
Figure. S6. Tyrosine-induced EMCDRs promote pulmonary metastasis niche.
Figure. S7. Tyrosinase agonist enhances excess melanosome dependent cell death (EMCD).
Figure. S8. Excess mobilization of tyrosinase impaired adhesion and chemotaxis associated gene expression.
Figure. S9. Schematic representation.

Supplementary figure legends

Figure S1

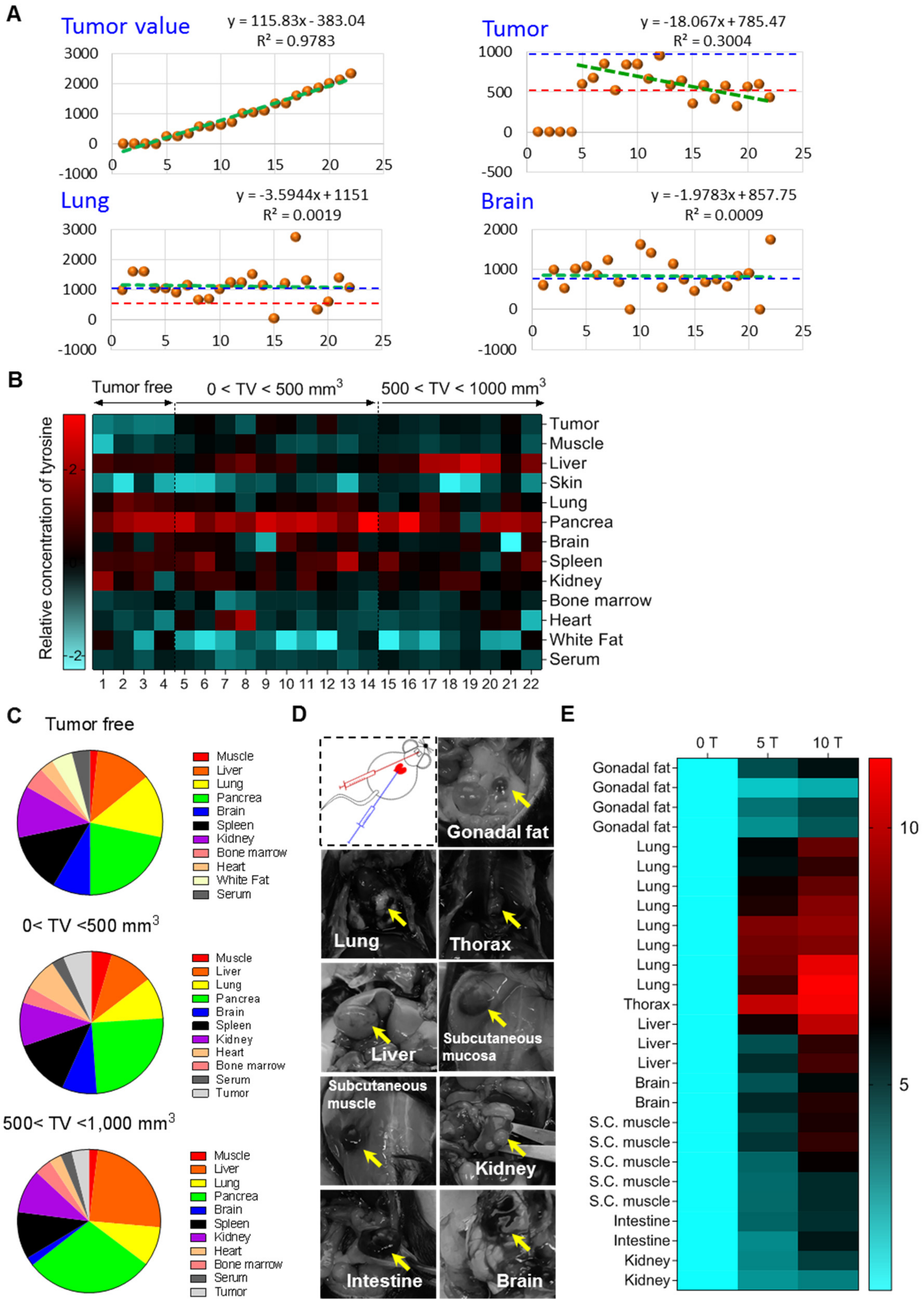

Figure S1. Tyrosine concentration in multiple organs have dynamic changes following tumor progression.

A. Normal B16 cells were injected into the mice subcutaneously. Tumor volume was recorded (mm^3). Tyrosine concentration in tumor, lung and brain were measured (ng/g tissue). Green line is calculated trend line, red line indicates the concentration at 500 ng/g, and blue line indicates the concentration at 1000 ng/g. **B.** Tyrosine contents in different tissues were presented by heatmap. **C.** Proportion chart of tyrosine content ratio in different tissues. The samples are grouped according to tumor size into tumor free, small tumor (0 < tumor volume < 500 mm^3) and large tumor (500 < tumor volume < 1000 mm^3). **D.** Photos of B16 tumors colonized in gonadal fat, lung, thorax, liver, subcutaneous mucosa, subcutaneous muscle, kidney, intestine and brain. B16 cells were injected into the heart and brain. **E.** Efficiency of tyrosine-based melanin synthesis in melanoma cells from different organs. Relative melanin production by 0 T, 5 T and 10 T tyrosine treatment in the melanoma cells isolated from above tissue localized tumors. S.C. indicates subcutaneous.

Figure S2

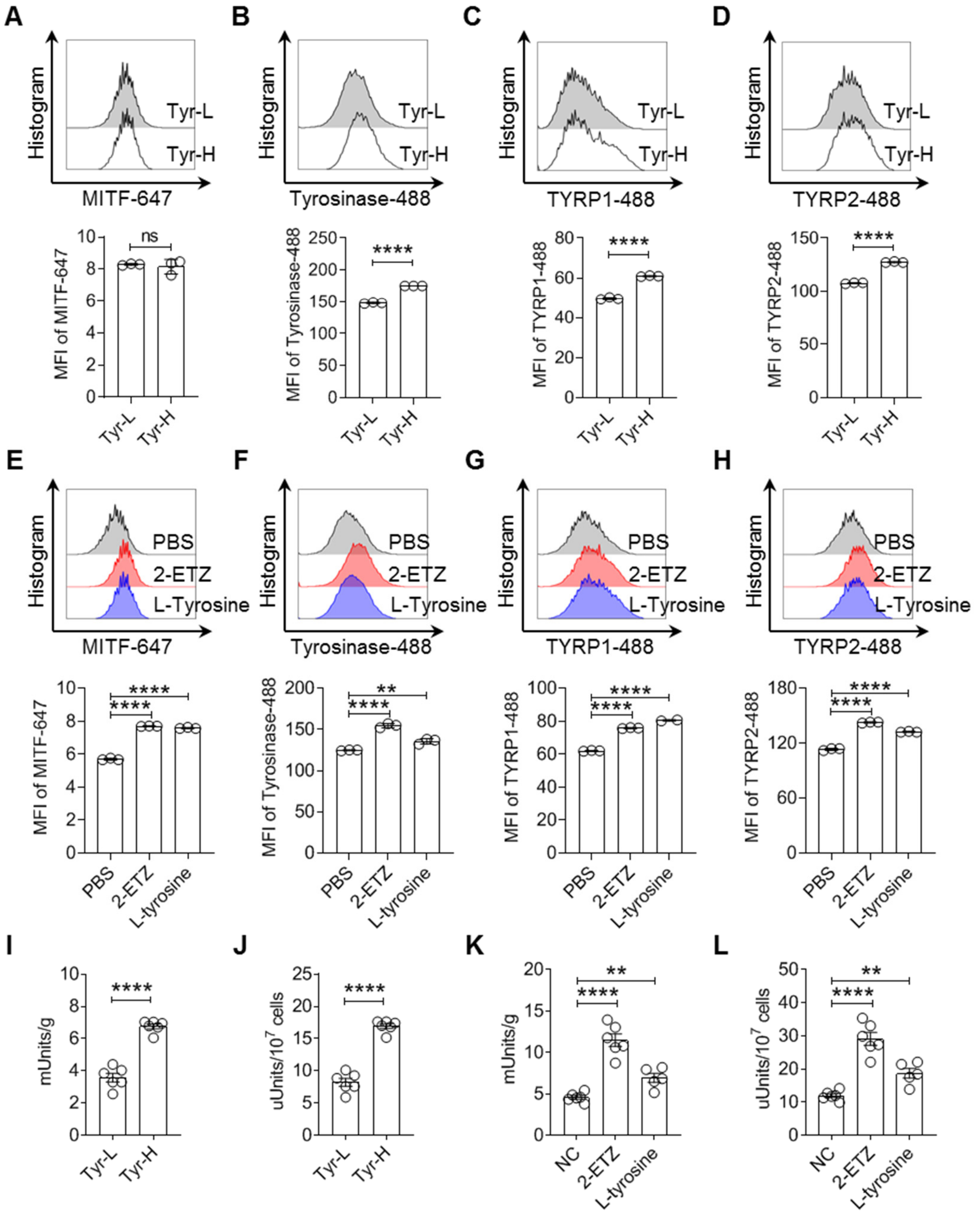

Figure S2. Expression of melanogenesis related proteins and tyrosinase activity.

A-D. Expression of MITF, Tyrosinase, TYRP1 and TYRP2 in Tyr-L B16 cells and Tyr-H B16 cells. **E-H.** Expression of MITF, Tyrosinase, TYRP1 and TYRP2 expression in normal B16 melanoma cells with or without treatment of 2-ETZ (250 μM) or L-tyrosine (5 T) for 24 h. **I-L.** Tyrosinase activity of Tyr-L B16 cells, Tyr-H B16 cells and normal B16 cells. Tyr-L B16 cells and Tyr-H B16 cells were cultured in standard DMEM culture medium. Normal B16 melanoma cells were treated with or without 2-ETZ (250 μM) or L-tyrosine (5 T) for 24 h. All data given as mean ± SEM., n ≥ 3. *$**P < 0.01$*, *$****P < 0.0001$*, ns, not significant. All results shown are representative of three independent experiments.

Figure S3

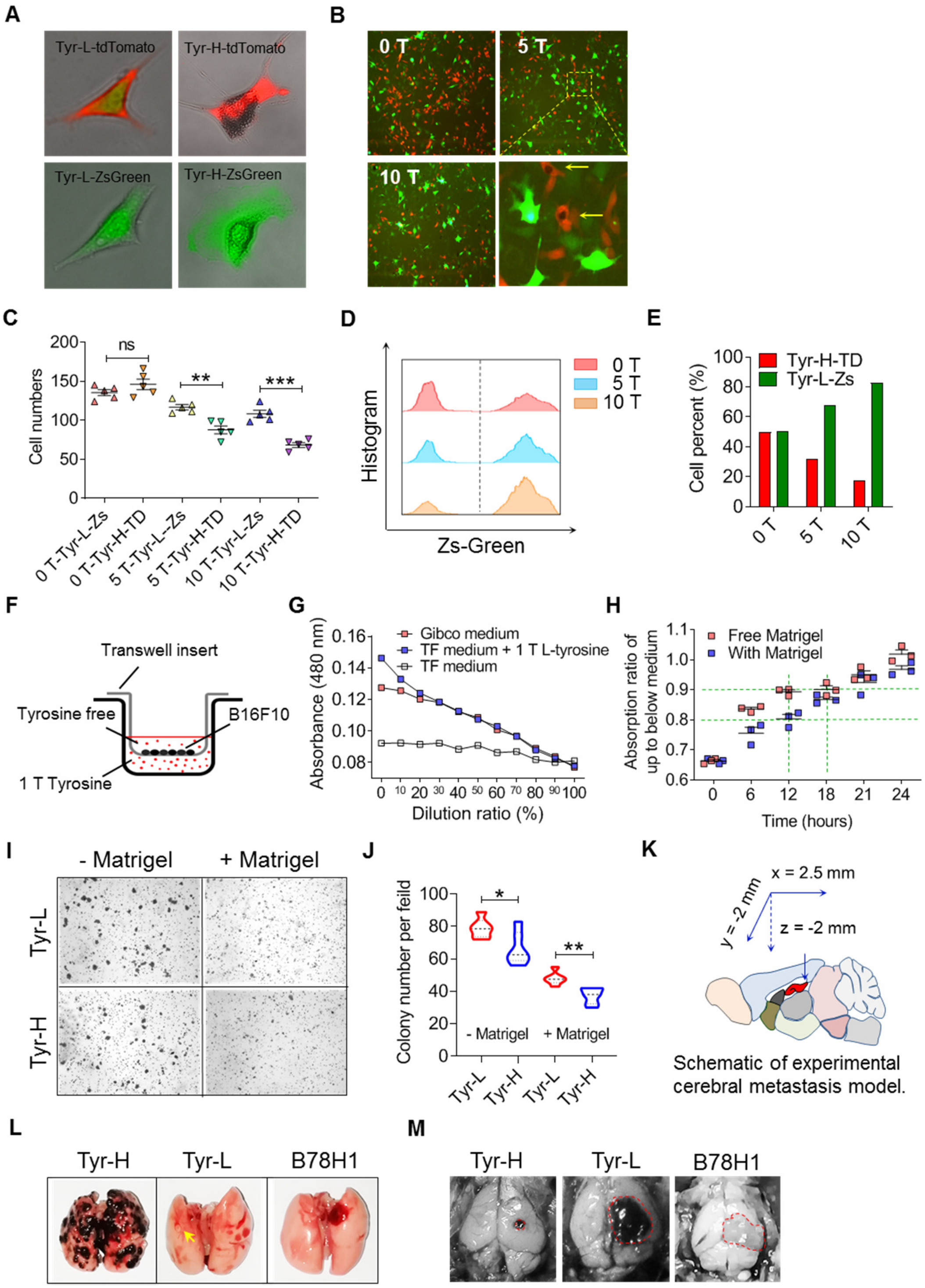
A
Tyr-L-tdTomato
Tyr-H-tdTomato
Tyr-L-ZsGreen
Tyr-H-ZsGreen
B
0 T
5 T
10 T
C
Cell numbers
ns
**
***
0 T-Tyr-L-Zs
0 T-Tyr-H-TD
5 T-Tyr-L-Zs
5 T-Tyr-H-TD
10 T-Tyr-L-Zs
10 T-Tyr-H-TD
D
Histogram
Zs-Green
0 T
5 T
10 T
E
Cell percent (%)
Tyr-H-TD
Tyr-L-Zs
0 T
5 T
10 T
F
Transwell insert
Tyrosine free
B16F10
1 T Tyrosine
G
Absorbance (480 nm)
Gibco medium
TF medium + 1 T L-tyrosine
TF medium
Dilution ratio (%)
H
Absorption ratio of up to below medium
Free Matrigel
With Matrigel
Time (hours)
I
- Matrigel
+ Matrigel
Tyr-L
Tyr-H
J
Colony number per feild
*
**
- Matrigel
+ Matrigel
Tyr-L
Tyr-H
Tyr-L
Tyr-H
K
x = 2.5 mm
y = -2 mm
z = -2 mm
Schematic of experimental cerebral metastasis model.
L
Tyr-H
Tyr-L
B78H1
M
Tyr-H
Tyr-L
B78H1

Figure S3. Tyrosine did not provide stronger chemotaxis for Tyr-H cells than Tyr-L cells.

A. Photos of tdTomato and ZsGreen labeled Tyr-L cells and Tyr-H cells. Cells were treated with 10 T tyrosine for 24 h. **B-C.** Photos and numbers of the mix co-cultured tdTomato labeled Tyr-H cells (Tyr-H-TD) and ZsGreen labeled Tyr-L cells (Tyr-L-Zs). Cells were treated with 5 T or 10 T tyrosine for 24 h. **D-E.** Flow cytometry analyzed percentage of tdTomato labeled Tyr-H cells (Tyr-H-TD) and ZsGreen labeled Tyr-L cells (Tyr-L-Zs). Cells were treated with 5 T or 10 T tyrosine for 24 h. **F.** Schematic showing the procedure of tyrosine chemotaxis detection assay by transwell. The upper chamber of transwell was loaded with tyrosine free DMEM culture medium (TF medium), and the lower chamber was loaded with the culture medium with 1 T tyrosine addition (1 T tyrosine). **G.** Tyrosine concentration change curve of the culture medium in different dilutions with water. Standard DMEM culture medium (Gibco) was the control for 1 T tyrosine culture medium. **H.** The tyrosine concentration ratio of the upper chamber to the lower chamber. Transwell was coated with or without matrigel. **I.** Photographs of migrated and infiltrated Tyr-L cells and Tyr-H cells. Transwell was coated with or without matrigel. **J.** Per field colony number of the above migrated and infiltrated Tyr-L cells and Tyr-H cells. **K.** Schematic of experimental cerebral metastasis model. **L-M.** Pulmonary (*i.v.* day 16) and cerebral colonization (*icv.* day 12) of Tyr-H cells, Tyr-L cells and B78H1 cells. All data given as mean ± SEM., n ≥ 3. $^{**}P < 0.01$, $^{***}P < 0.001$, ns, not significant. All results shown are representative of three independent experiments.

Figure S4

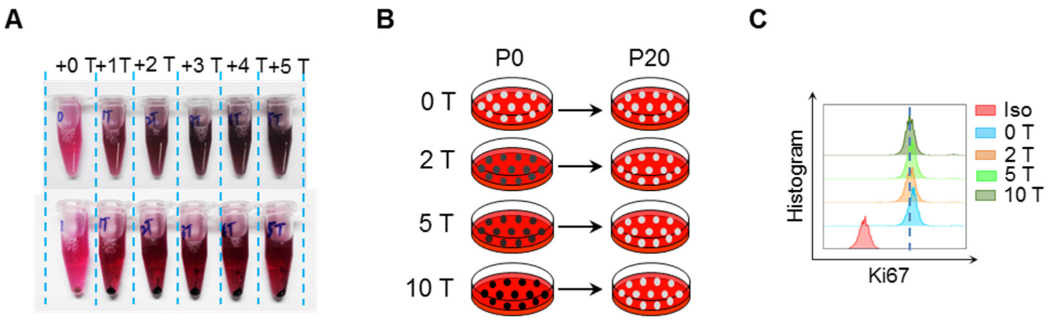

Figure S4. Excess tyrosine enriches Tyr-L B16 cells.

A. Photos of B16 cells with different concentration of tyrosine treatment. The upper row and the lower row respectively indicate before and after centrifugation. **B.** Enrichment of tyrosine insensitive B16 cells. B16 cells were cultured in the conditional medium with different concentration of tyrosine for more than 20 passages. All cells will turn back to be amelanotic when they were passaged for more than 20 generations in the conditional culture medium. **C.** Cell proliferation was analyzed by Ki67 staining. B16 cells were cultured for more than 20 passages in the conditional medium with 2 T, 5 T and 10 T tyrosine.

Figure S5

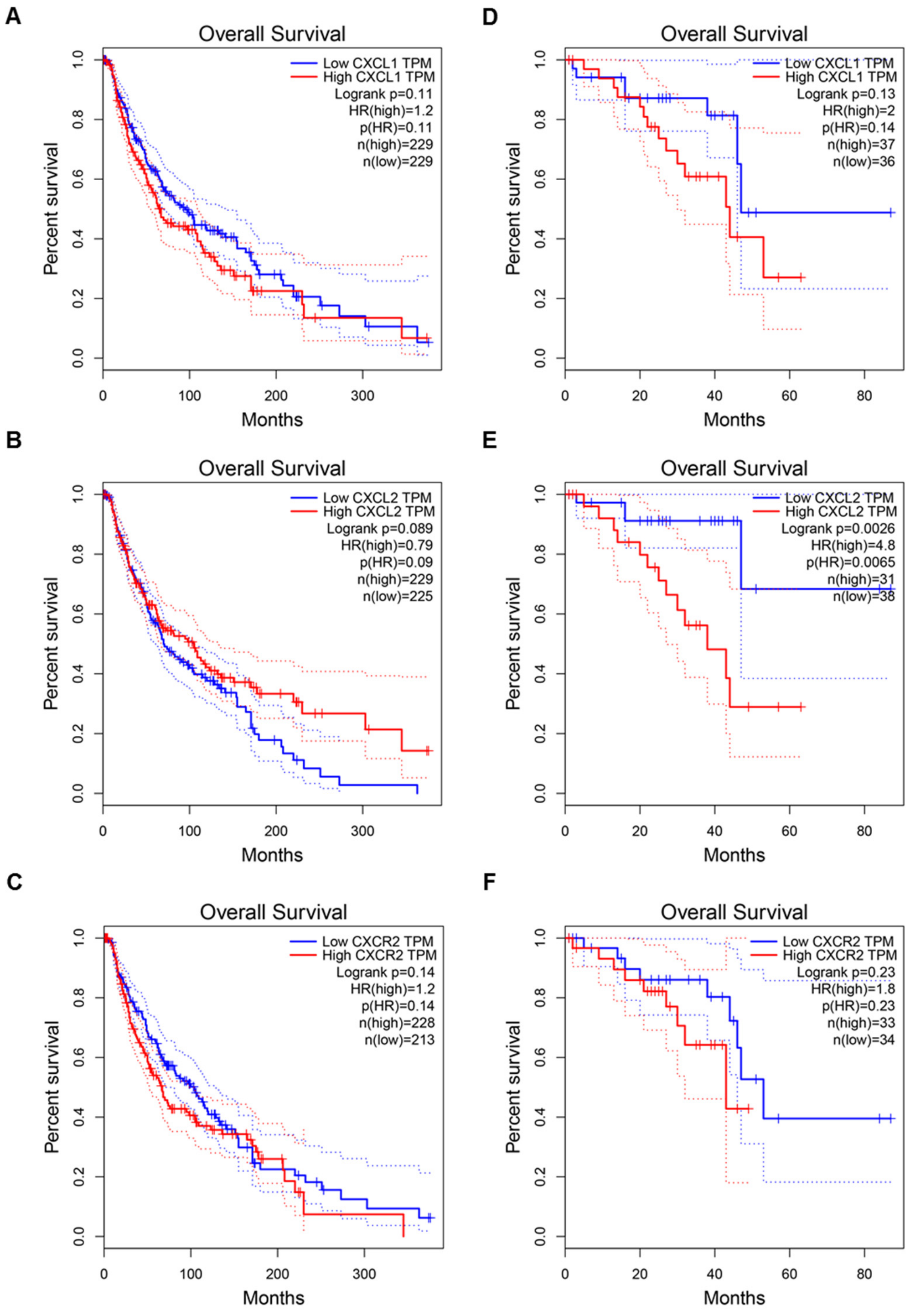

Figure S5. High level expression of CXCL1, CXCL2 and CXCR2 predicts poor prognosis of melanoma.

A-F. The survival probability of skin cutaneous melanoma (SKMC, A, B, C) and uveal melanoma (UVM, D, E, F) patient derived melanoma tumor with higher and lower *CXCL1*, *CXCL2* and *CXCR2* expression. These data acquired from the TCGA and the GTEx projects, using a standard processing pipeline by GEPIA website.

Figure S6

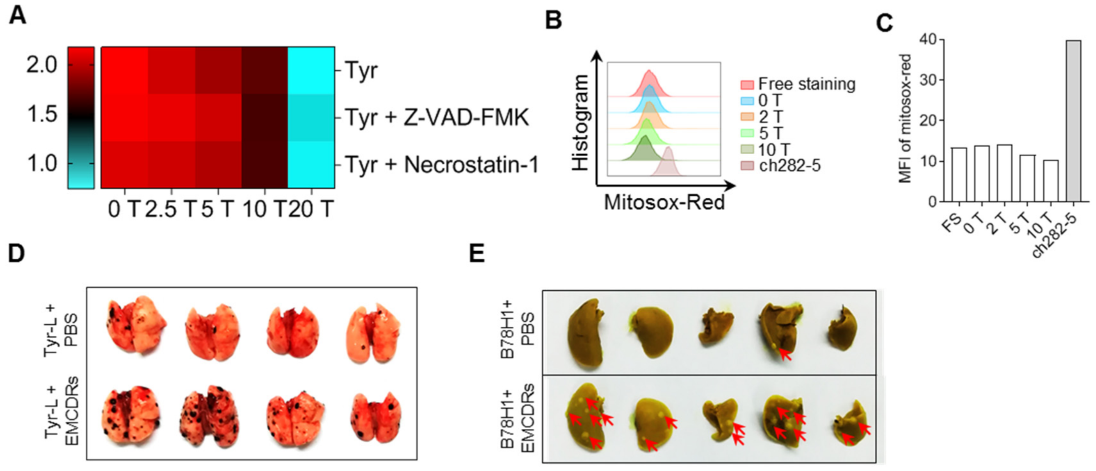

Figure S6. Tyrosine-induced EMCDRs promote pulmonary metastasis niche.

A. Relative cell viability of B16 cells. B16 cells were treated with tyrosine and pan-caspase inhibitor Z-VAD-FMK or RIP1 inhibitor necrostatin-1 for 24 h. **B-C.** B16 cells were treated with tyrosine or ch282-5 for 24 h, then the mitochondrial ROS was measured by the mitosox-red staining. **D-E.** Photos of the Tyr-L cell (D) or B78H1 cell (E) invasive lung. Tyr-L cells mixed with EMCDRs were transplanted into the mice through *i.v.* injection.

Figure S7

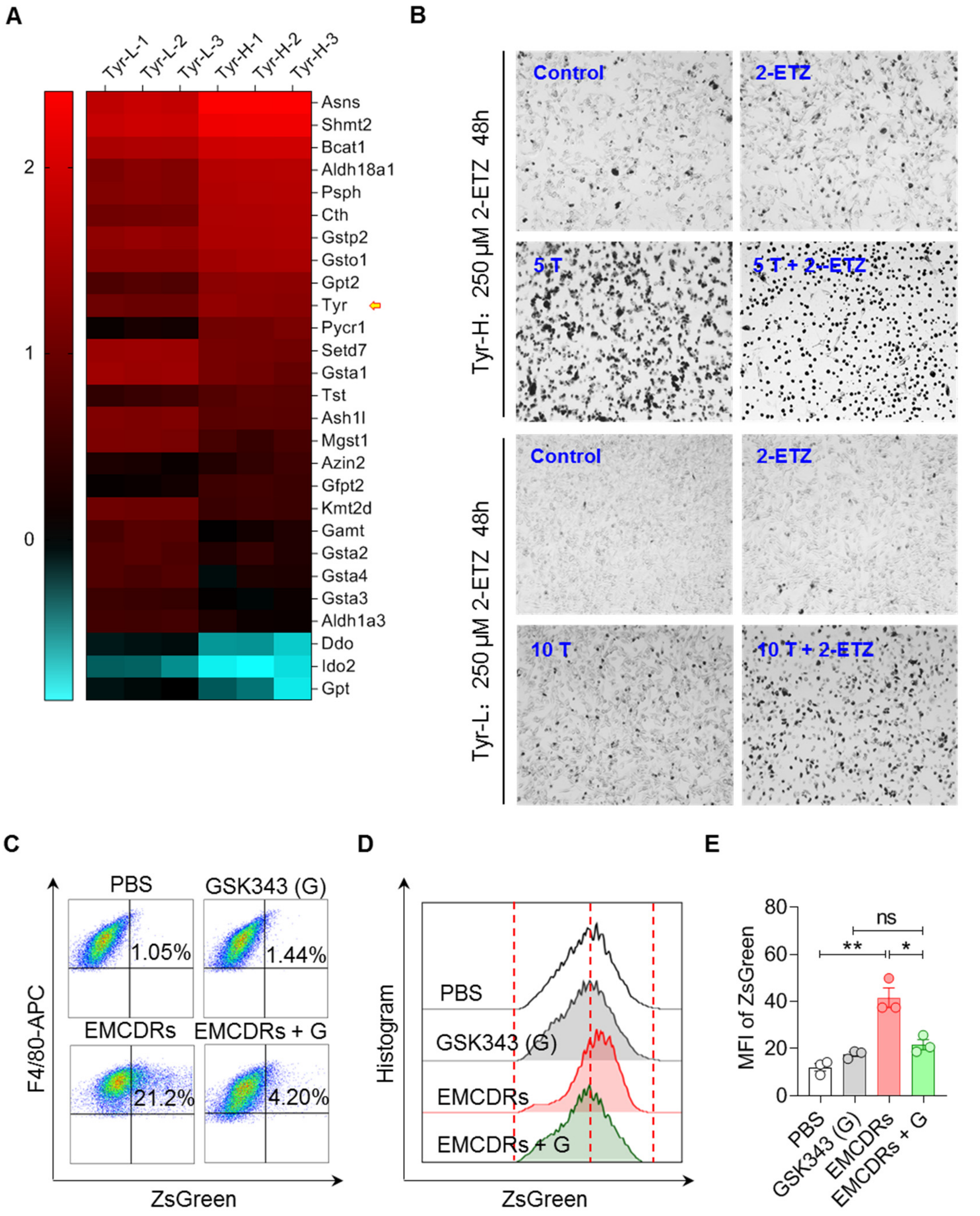

Figure S7. Tyrosinase agonist enhances excess melanosome dependent cell death (EMCD).

A. Differentially expressed genes associated with amino acid metabolism were enriched by KEGG analysis and presented by heatmap. **B.** Photos of Tyr-H cells and Tyr-L cells. Cells were treated by tyrosine combining with 2-Ethoxybenzamide (2-ETZ) for 48 h. **C-E.** Peritoneal macrophages were co-cultured with EMCDRs with or without 40 µM GSK343 addition for 10 h. Macrophage phagocytosis were detected by flow cytometry. All data given as mean ± SEM., n ≥ 3. *$P < 0.05$, **$P < 0.01$, ns, not significant. All results shown are representative of three independent experiments.

Figure S8

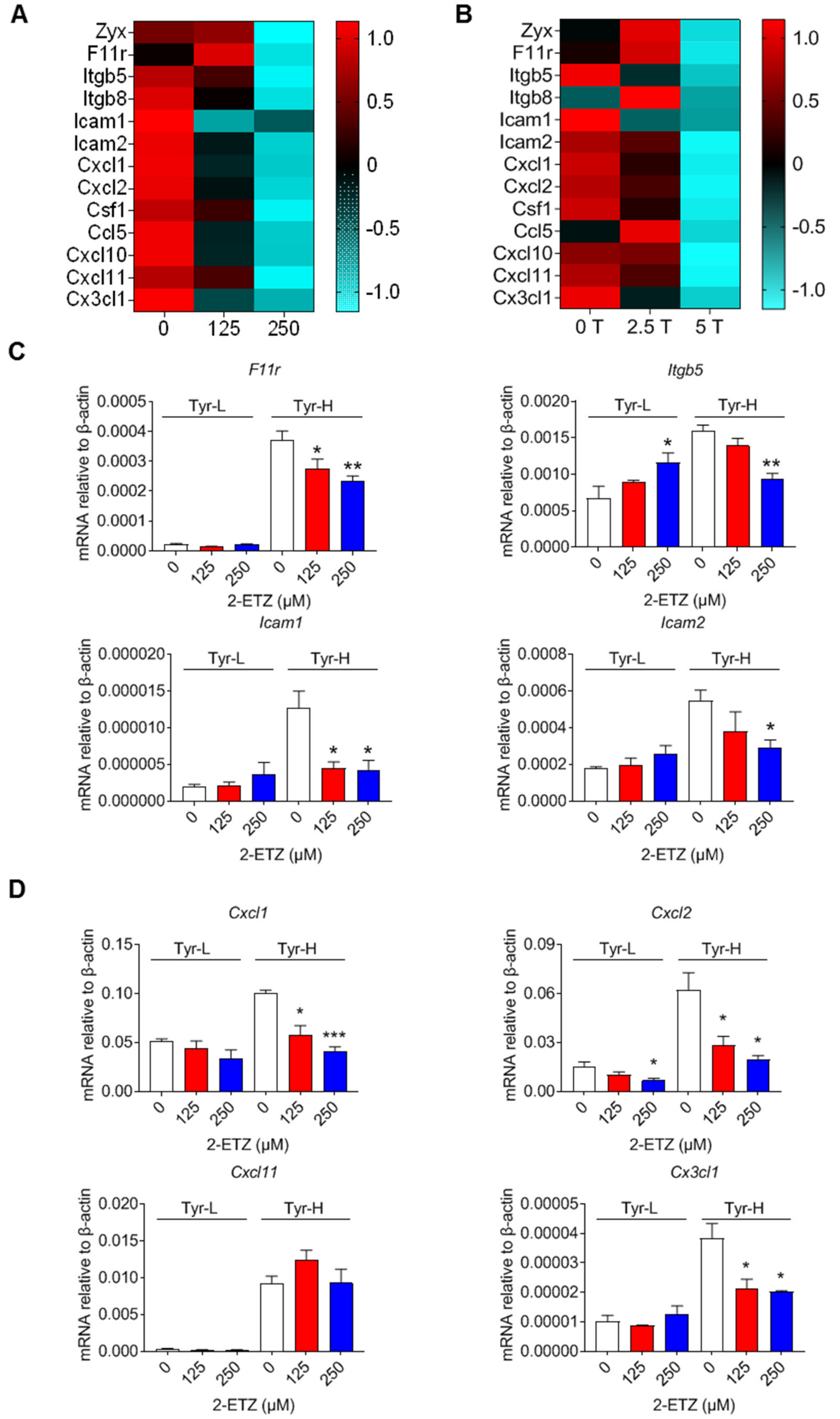
A
Zyx
F11r
Itgb5
Itgb8
Icam1
Icam2
Cxcl1
Cxcl2
Csf1
Ccl5
Cxcl10
Cxcl11
Cx3cl1
0
125
250
1.0
0.5
0
-0.5
-1.0
B
0 T
2.5 T
5 T
C
F11r
Itgb5
Icam1
Icam2
D
Cxcl1
Cxcl2
Cxcl11
Cx3cl1
mRNA relative to β-actin
Tyr-L
Tyr-H
2-ETZ (μM)

Figure S8. Excess mobilization of tyrosinase impaired adhesion and chemotaxis associated gene expression.

A-B. Adhesion and chemotaxis associated gene expression in B16 cells. B16 cells were treated with 0 μM, 250 μM, 500 μM 2-Ethoxybenzamide (2-ETZ, A), or 0 T, 2.5 T, 5 T tyrosine (B) for 24 h. **C-D.** Adhesion and chemotaxis associated gene expression in Tyr-L cells and Tyr-H cells. Tyr-L cells and Tyr-H cells were treated with 0 μM, 125 μM, 250 μM 2-Ethoxybenzamide (2-ETZ) for 24 h. All data given as mean ± SEM., n ≥ 3. *$P < 0.05$, **$P < 0.01$, ***$P < 0.001$, ns, not significant. All results shown are representative of three independent experiments.

Figure S9

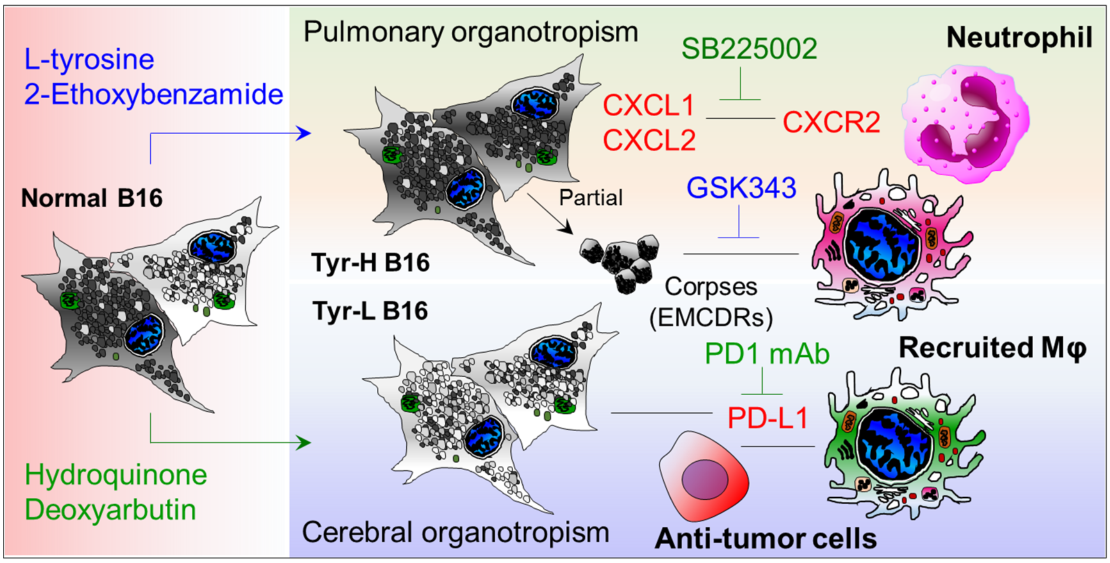

Figure S9. Schematic representation. Tyr-H cells efficiently use tyrosine to synthesize melanin. At an appropriate concentration of tyrosine, Tyr-H cells secrete large amounts of CXCL1, CXCL2 and other chemokines to recruit neutrophils and macrophages (Mφ) to the lung. Some extremely sensitive B16 cells die from excessive melanosome accumulation, and the remains of dead cells (EMCDRs) train macrophages to promote the metastatic microenvironment and lung metastases. Tyr-L cells inefficiently use tyrosine to synthesize melanin. Tyr-L cells induce macrophages to express PD-L1 and promote their colonization in the brain. On the one hand, 2-ETZ plus tyrosine over-stimulates melanin synthesis promote the death of Tyr-H cells, simultaneously inhibiting the phagocytosis of macrophages suppresses lung metastasis microenvironment, combined with PD1 mAb to decrease melanoma metastasis to the lung and brain. On the other hand, hydroquinone inhibits melanin synthesis in Tyr-H cells, which allows Tyr-H cells to survive in the lung, simultaneously disturbing the recruitment of neutrophils and macrophages with SB225002 to inhibit lung metastasis microenvironment, combined with PD1 mAb suppresses the metastasis to the lung and brain.
